# eNOS plays essential roles in the developing heart and aorta linked to disruption of Notch signalling

**DOI:** 10.1242/dmm.050265

**Published:** 2024-01-22

**Authors:** Lorraine Eley, Rachel V. Richardson, Ahlam Alqahtani, Bill Chaudhry, Deborah J. Henderson

**Affiliations:** Bioscience Institute, Newcastle University, Centre for Life, Central Parkway, Newcastle upon Tyne, NE1 3BZ, UK

**Keywords:** eNOS, Congenital heart defects, Cartilaginous dysplasia, Baroreceptors, Notch

## Abstract

eNOS (NOS3) is the enzyme that generates nitric oxide, a signalling molecule and regulator of vascular tone. Loss of eNOS function is associated with increased susceptibility to atherosclerosis, hypertension, thrombosis and stroke. Aortopathy and cardiac hypertrophy have also been found in eNOS null mice, but their aetiology is unclear. We evaluated eNOS nulls before and around birth for cardiac defects, revealing severe abnormalities in the ventricular myocardium and pharyngeal arch arteries. Moreover, in the aortic arch, there were fewer baroreceptors, which sense changes in blood pressure. Adult eNOS null survivors showed evidence of cardiac hypertrophy, aortopathy and cartilaginous metaplasia in the periductal region of the aortic arch. Notch1 and neuregulin were dysregulated in the forming pharyngeal arch arteries and ventricles, suggesting that these pathways may be relevant to the defects observed. Dysregulation of eNOS leads to embryonic and perinatal death, suggesting mutations in eNOS are candidates for causing congenital heart defects in humans. Surviving eNOS mutants have a deficiency of baroreceptors that likely contributes to high blood pressure and may have relevance to human patients who suffer from hypertension associated with aortic arch abnormalities.

## Teaser

**Editor's choice:** eNOS is essential during cardiogenesis and null mutants frequently die during embryogenesis or at birth. Cardiovascular defects in mildly affected adult survivors can be traced back to intrauterine life.

## INTRODUCTION

**Fig. 1.**
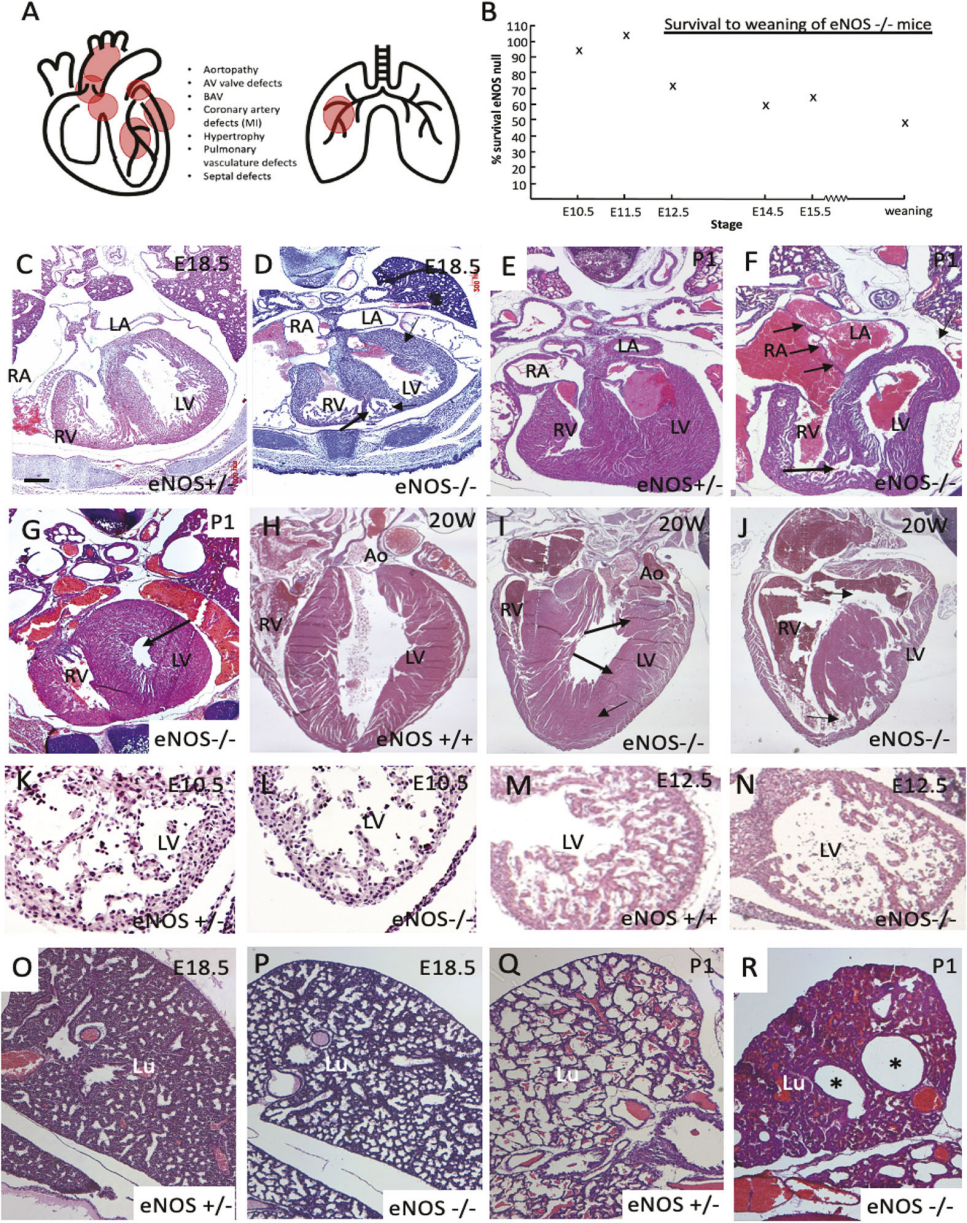
**Structural pulmonary and cardiovascular defects in neonatal eNOS null mice.** (A) Cartoon showing sites of reported defects in heart and lungs in eNOS null mice. (B) Graph showing the overall survival of homozygous mutants at different stages of development and maturity. Percentage survival is based on genotyping of pups from heterozygote crosses prenatally, but on observed death of pups postnatally. (C-G) Four chamber view images of eNOS^+/−^ and eNOS^−/−^ neonates at E18.5 and P1 (*n*=7 at each stage for controls and nulls). The mutant hearts are abnormally shaped, the right ventricular myocardium is thinned (arrows in D) and muscular ventricular septal defects can be seen in some cases (long arrow in F). In some eNOS nulls, the left ventricular cavity is very small (arrow in G). (H-J) Ventricular defects are seen in eNOS null adults, with hypertrophy of the left ventricular wall (arrows in I) and ventricular septal defects (arrows in J). (K-N) Defects in the ventricular myocardium are becoming apparent at E10.5 (*n*=6 for controls and nulls) and at E12.5 (*n*=3 for controls and nulls). Reduced trabeculation can be seen in the ventricles at both stages. (O-R) Lung morphology in controls and eNOS nulls at E18.5 and P1 (*n*=7 at each stage for controls and nulls). Before breathing commences, the lungs appear similar in both controls and mutants (O,P). After birth and the onset of breathing, the airways are expanded in control animals (Q) but remain generally underinflated with unexpanded airways in eNOS nulls (R). However, there are also hyperexpanded regions (asterisks in R). Ao, aorta; LA, left atrium; Lu, lung; LV, left ventricle; RV, right atrium; RV, right ventricle. Scale bar: 400 µm in C,D; 440 µm in E-G; 800 µm in H-J; 65 µm in K,L; 115 µm in M,N; 1300 µm in O-R.

Endothelial nitric oxide synthase (eNOS; NOS3) is an enzyme that is required for the production of nitric oxide, an important signalling molecule and potent regulator of vascular tone (Zhao et al., 2015). From early in development, eNOS is produced in endothelial cells and the endocardium of the heart (Teichert et al., 2008), and is maintained in the adult (Gregg et al., 1998). It is also expressed at lower levels in other cell types, including cardiomyocytes (Wei et al., 1996; Feron et al., 1996). eNOS null mice are known to exhibit endothelial dysfunction, which manifests as hypertension and increased susceptibility to thrombosis, atherosclerosis and stroke (reviewed by Atochin and Huang, 2010). More recently, it has been suggested that eNOS null mice have features of aortopathy and cardiac hypertrophy (Peterson et al., 2020) (Fig. 1A). It has also been reported that ∼30% of eNOS null mice have a bicuspid aortic valve (Lee et al., 2000; Feng et al., 2002; Fernández et al., 2009; Peterson et al., 2018, 2020). Moreover, some eNOS null pups die in the neonatal period with septal defects and cardiac hypertrophy (Feng et al., 2002; Liu and Feng, 2012). Defects in the formation of the coronary vasculature have also been linked to myocardial infarction in newborn eNOS null pups (Liu et al., 2014), and pulmonary abnormalities have been described in eNOS mice at the time of birth (Han et al., 2004). Thus, there are a broad range of pathologies that have been reported to appear in the absence of eNOS, noted both in the adult and in the perinatal period, although relatively little is known about the aetiology of any congenital abnormalities.

In order to address the issue of whether eNOS is broadly required during the development of the cardiovascular system, we evaluated eNOS null fetuses and neonates for cardiac defects, observing a high proportion of abnormalities in the ventricular myocardium and pharyngeal arch arteries at both time points. Moreover, baroreceptors were lost from the aortic arch. Investigation of adult eNOS null survivors revealed evidence of cardiac hypertrophy and aortopathy, and cartilaginous metaplasia specifically localised to the peri-ductal region where the aortic arch joins the descending aorta. The origins of all of these defects could be traced to before birth. Notch1 and its ligand Jag1 were disrupted in specific regions of the forming ventricles and pharyngeal arch arteries, concurrent with the first appearance of defects. Thus, it appears that eNOS plays important roles during embryonic and fetal development, at least in part through its regulation of Notch signalling.

## RESULTS

As many different pathologies that potentially lead to death have been described in the eNOS null mutant mouse (Fig. 1A), we first looked at our colony to look at the relative contributions from the different abnormalities.

### Most eNOS null pups die before birth or in the neonatal period, with defects in the ventricular myocardium

Our eNOS colony and other mice are maintained on the Charles River C57Bl/6J background and regularly backcrossed to maintain vigour. We maintained an experimental colony where all eNOS animals were homozygous null mutants. The observed mortality rate in the eNOS null colony during the first week of life was 23% (96/412 null pups died). This is likely to be an under-representation of the true mortality rate, as some null pups that died shortly after birth will have been immediately eaten by the mother and could not be included in this analysis. However, this is an excess mortality when compared with an observed mortality rate of 10% in our eNOS heterozygote population and 8% in the pre-weaning period in other transgenic mice on the same genetic background, e.g. Mef2c-AHF-Cre (Verzi et al., 2005). Beyond this, and before 20 weeks of age, there was a low attrition and only 10 out of the 316 null adults (3%) died in this interval. As multiple causes of neonatal death have been described, we evaluated a small group of null animals that we collected after death in the immediate neonatal period (P1-P2) and some collected at the end of gestation (E17.5-E18.5).

Intra-cardiac defects were observed in five out of seven late gestation fetuses and all seven of the neonatal eNOS null animals examined. A reproducible set of defects was identified, although the severity was variable (Fig. 1C-G, Table 1). The most striking and consistent defects in eNOS null mutants were found in the ventricular myocardium, which was thinned at the apex of the right ventricle (Fig. S1). Moreover, the trabeculae appeared abnormal in both ventricles in eNOS mutants, when compared with controls (Fig. 1D,F,G; Fig. S1). The intraventricular septum was affected in a similar way to the rest of the ventricular myocardium, resulting in muscular ventricular septal defects (Fig. 1F; Fig. S1). Although myocardial infarction has been reported in 50% of P0-P5 eNOS null pups previously (Liu et al., 2014), we saw no evidence of this in any of the late fetal and neonatal animals examined in this study (0/13) or in any others by external examination (more than 30 neonatal hearts). Formation of the atrial septum appeared normal, however, in three out of seven postnatal hearts the atria appeared dilated and were engorged with blood, in keeping with heart failure (Fig. 1F; Fig. S1). Bicuspid aortic valves were observed in four out of 18 (22%) animals, but although previous studies (Liu et al., 2013) had suggested that the atrioventricular valves were abnormal, this did not appear to be the case in our colony (Fig. S2). There did not appear to be any correlation between BAV and the severity of the other cardiovascular defects (Table 1).

**Table 1.**
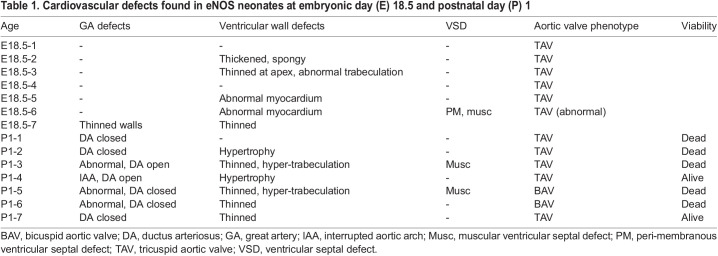
Cardiovascular defects found in eNOS neonates at embryonic day (E) 18.5 and postnatal day (P) 1

We expected that eNOS null mice that survive to adulthood would carry the fewest congenital cardiac abnormalities of the eNOS null population. We therefore evaluated the hearts of 11 20-week-old, apparently healthy (although one was small for age), eNOS null mice for abnormalities in the structure of the ventricles. Externally, there were no obvious signs of cardiac hypertrophy or of myocardial infarction. However, sectioning these hearts revealed abnormalities in six out of 11 of these hearts (Fig. 1H-J), including three with peri-membranous VSD, one with muscular VSD and four with septal hypertrophy with small left ventricular lumen.

We were interested to know at what stage of development the ventricular myocardium abnormality first became apparent in eNOS null animals. An analysis of in-crosses of eNOS heterozygous mice suggested that some eNOS nulls were dying *in utero*, from ∼E12.5 of gestation (Fig. 1B). We therefore examined null embryos in the preceding days of development and found abnormalities in formation of the trabecular layer in both ventricles (*n*=6) as early as E10.5-E11.5 (Fig. 1K,L). Notably, there were fewer and shorter trabeculae, and this failure to form a normal trabecular network was even more apparent at E12.5 (Fig. 1M,N).

### Pulmonary malformations are observed after birth

Deaths of eNOS null mice in the perinatal period have been ascribed to pulmonary malformations (Han et al., 2004), so we examined seven eNOS null pups at E17.5-E18.5, immediately before birth. In a masked experiment, it was not possible to distinguish the null lungs from stage-matched controls (Fig. 1O,P). In contrast, in six eNOS null mice that died immediately after birth, and a seventh null animal that was alive, examination of the lungs indicated that in six out of seven cases (including the live eNOS null pup) there was compacted lung structure, indicating poor inflation (Fig. 1Q,R). Most strikingly, however, there were greatly expanded alveoli in four out of seven eNOS null neonates (Fig. 1R), again including the live animal. Thus, lung abnormalities were common in our eNOS null pups but were apparent only after birth when breathing commenced. We were not able to conclude whether the pulmonary abnormalities were sufficient to cause or contribute to death.

Taken together, these observations indicate that abnormal ventricular wall development involving both the compact and trabecular layer is likely to be a major cause of death during mid-gestation (Conway et al., 2003). The transition to extrauterine circulation and breathing at the time of birth, is a later point at which myocardial and lung abnormalities are likely to result in death. The intracardiac abnormalities found in surviving animals might ultimately result in premature death.

### The thoracic great arteries are structurally abnormal in eNOS null mice

**Fig. 2.**
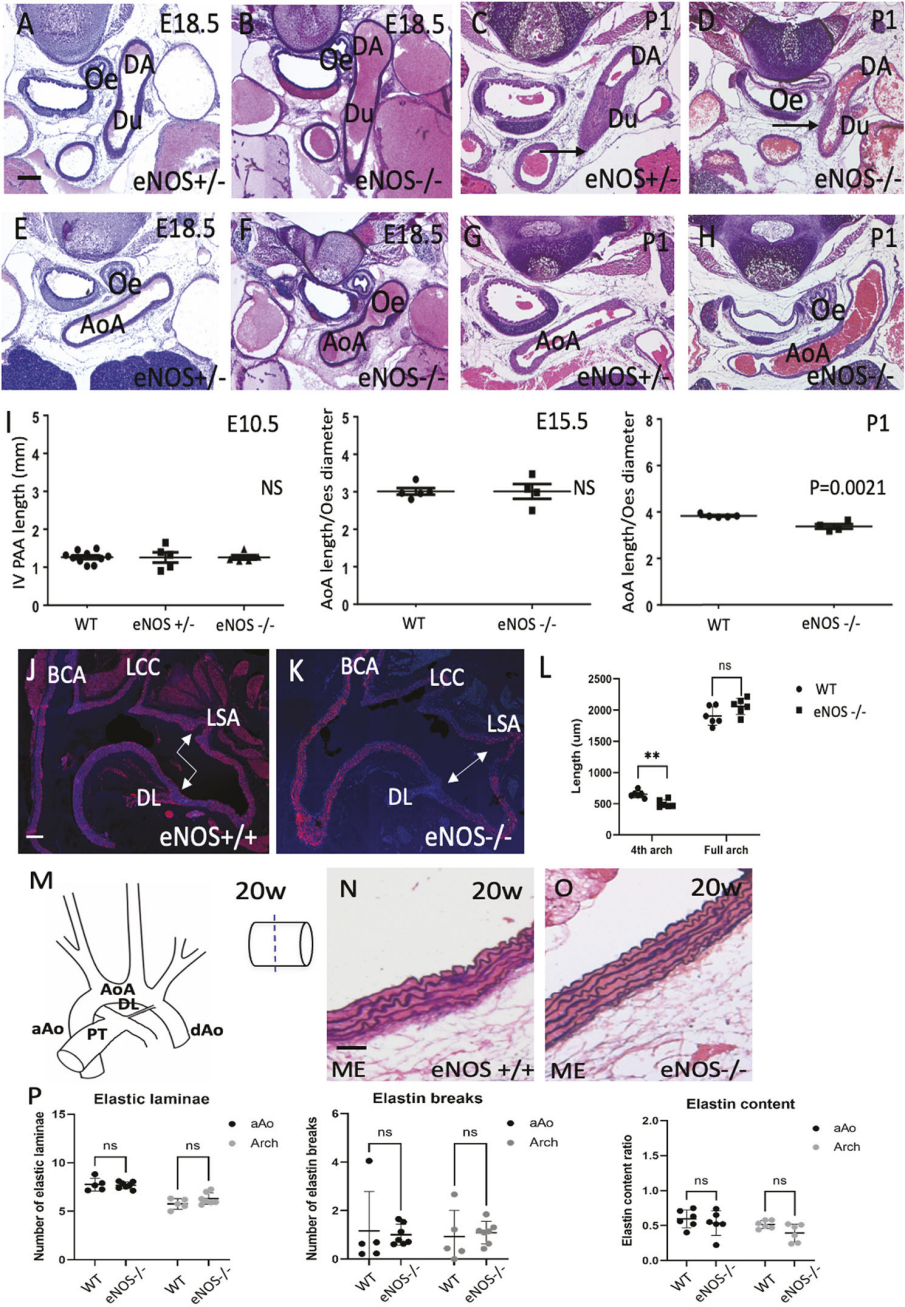
**Aortic arch abnormalities in eNOS null fetuses and adults.** (A-H) The ductus arteriosus looks grossly normal in eNOS nulls at E18.5 but remains patent in some neonates at P1 [arrow in D; compare with closed duct in control in C (arrow); *n*=7 at each stage for controls and nulls]. (E-H) The aortic arch is enlarged and tortuous in eNOS mutants both at E18.5 and at P1, compared with wild type or heterozygote controls. (I) Measurements of the 4th pharyngeal arch artery (aortic arch) show that, although there is no difference between controls and eNOS nulls at E10.5 and E15.5, the arch is shorter in nulls by P1. *n*≥5 for each genotype at each stage. The E15.5 and P1 arch measurements were normalised to oesophagus size to take into account any differences in the age and thus overall size of the fetuses. No differences in oesophagus size were found between controls and eNOS nulls. Data are mean±s.e.m. (J-L) Ascending and transverse aorta, with associated aortic arch arteries, in eNOS wild type (J) and mutant (K). Double-headed arrows in J,K indicate that, whereas the LSA is proximal to the DL in wild-type animals, they lie opposite one another in the eNOS nulls (*n*=6 for each genotype). Graph in L shows that, although there is no difference between the overall length of the aortic arch (from the BCA to LSA) between wild types and eNOS nulls, the distance between the LCC and the DL is significantly shorter in the mutants (see Fig. S3D for image showing where these measurements were taken). **P<0.01. Data are mean±s.d. (M) Cartoon showing the thoracic aortic tree. (N,O) Miller's elastin (ME) staining showing elastic laminae in the aortic arch of control and eNOS nulls at 20 weeks of age. (P) Quantification of the numbers of elastic laminae, elastin breaks and total elastin content in the ascending aorta (black dots) and aortic arch (grey dots), showing that there are no statistical differences between 20-week-old controls and eNOS nulls for any of these parameters. *n*≥5 for each genotype. Data are mean±s.d. aAo, ascending aorta; AoA, aortic arch; BCA, brachiocephalic artery; DA/dAO, descending aorta; Du, ductus arteriosus; DL, ductal ligament; LCC, left common carotid artery; LSA, left subclavian artery; ME, Miller's elastin; Oe, oesophagus; PT, pulmonary trunk. Scale bars: 600 µm in A,B,E,F; 660 µm in C,D,G,G; 200 µm in J,K; 50 µm in N,O.

Highly penetrant aortopathy has been described in adult eNOS null mice (Peterson et al., 2020). We therefore carefully examined the thoracic aortic tree of our eNOS null mutants at different stages of development to identify newly identified and previously described features. In the perinatal period (E18.5 and P1-P2) the majority of eNOS nulls examined exhibited aortic arch arteries with either a tortuous appearance (three out of seven) or with an interrupted aortic arch (one out of seven) (Fig. 2A-H). Most animals had the expected left dominant arch pattern but in two out of seven examined at E18.5 there was a right-dominant aortic arch. Measurements confirmed that the aortic arch was shorter in eNOS nulls at the time of birth compared with matched controls (*P*=0.0021; Fig. 2I), with narrowing of the aortic arch close to the descending aorta in five out of seven null pups examined (Fig. 2C,D,G,H). The ductus arteriosus had closed normally in all of the age-matched wild-type controls and in five out of seven eNOS null neonates, although it remained open in two out of seven, including one animal that was collected while still alive (Fig. S3A). In view of the tortuosity, we measured the 4th and 6th aortic arch arteries in E10.5 and E15.5 null embryos (see Fig. S3). At these stages, the vessels were of normal appearance and also of normal length (Fig. 2I; Fig. S3E,F). In adult eNOS mice at 20 weeks of age, the length of the transverse arch of the aorta, between the brachiocephalic and left subclavian arteries (see Fig. S3), was similar in wild-type and eNOS null animals (Fig. 2J-L), although the positioning of the ductal ligament relative to that of the left subclavian artery was altered in the mutant animals (double-headed arrows in Fig. 2J,K). Specifically, the LSA was proximal to the ductal ligament in all six wild-type animals examined, whereas the two arteries were located in the same region of the arch in all six of the eNOS null animals (Fig. 2J,K). Moreover, the distal aortic arch, which lies between the left common carotid and the left subclavian arteries, and corresponds to the region derived from the left 4th pharyngeal arch artery (Molin et al., 2002), was significantly shorter in eNOS mutants compared with wild types (Fig. 2L).

As aortopathy frequently evolves during postnatal life, we examined 20-week-old eNOS null animals. Overall, the histological appearances were remarkably normal and we found no evidence of impending aneurismal dilation or dissections, as had been reported previously (Peterson et al., 2020). Abnormalities of elastin, including fragmentation, are associated with aortopathy in eNOS null mice (Bonderman et al., 1999; Tadros et al., 2009). We evaluated both the numbers of elastin layers and also the complexity of the elastin network in the ascending aorta, the aortic arch and the descending aorta in eNOS null animals at 20 weeks of age (Fig. 2M-P). These analyses revealed no statistical differences with regard to numbers of elastic laminae, elastin breaks or overall elastin content in the ascending aorta and aortic arch in eNOS null compared with wild-type animals. Alcian Blue histological stain was also used to delineate the extent of extracellular matrix in the same regions but no differences were found between 20-week-old eNOS mice compared with controls (Fig. S3G,H). Thus, in contrast to previous studies (Peterson et al., 2020), we did not find overt evidence of aortopathy in our eNOS null mice.

To uncover latent aortopathy, angiotensin II (0.8 g/kg bodyweight/day) was infused over several days by subcutaneous mini-osmotic pump (Patel et al., 2015). This potent inducer of hypertension (Patelis et al., 2017) resulted in aortic dissection (in four out of five animals) and gross thoracic aortic aneurysms (in one out of five animals) affecting the ascending aorta and aortic arch; none of these effects were seen in vehicle-treated eNOS null mice (Fig. S3I-M). Taken together, these studies indicate that the structural resilience of the aortic wall is abnormal, as evidenced by fetal tortuosity and susceptibility to aneurysm formation with induced hypertension.

### eNOS null mice have reduced smooth muscle marker expression and develop cartilaginous metaplasia restricted to the periductal region of the aortic arch

**Fig. 3.**
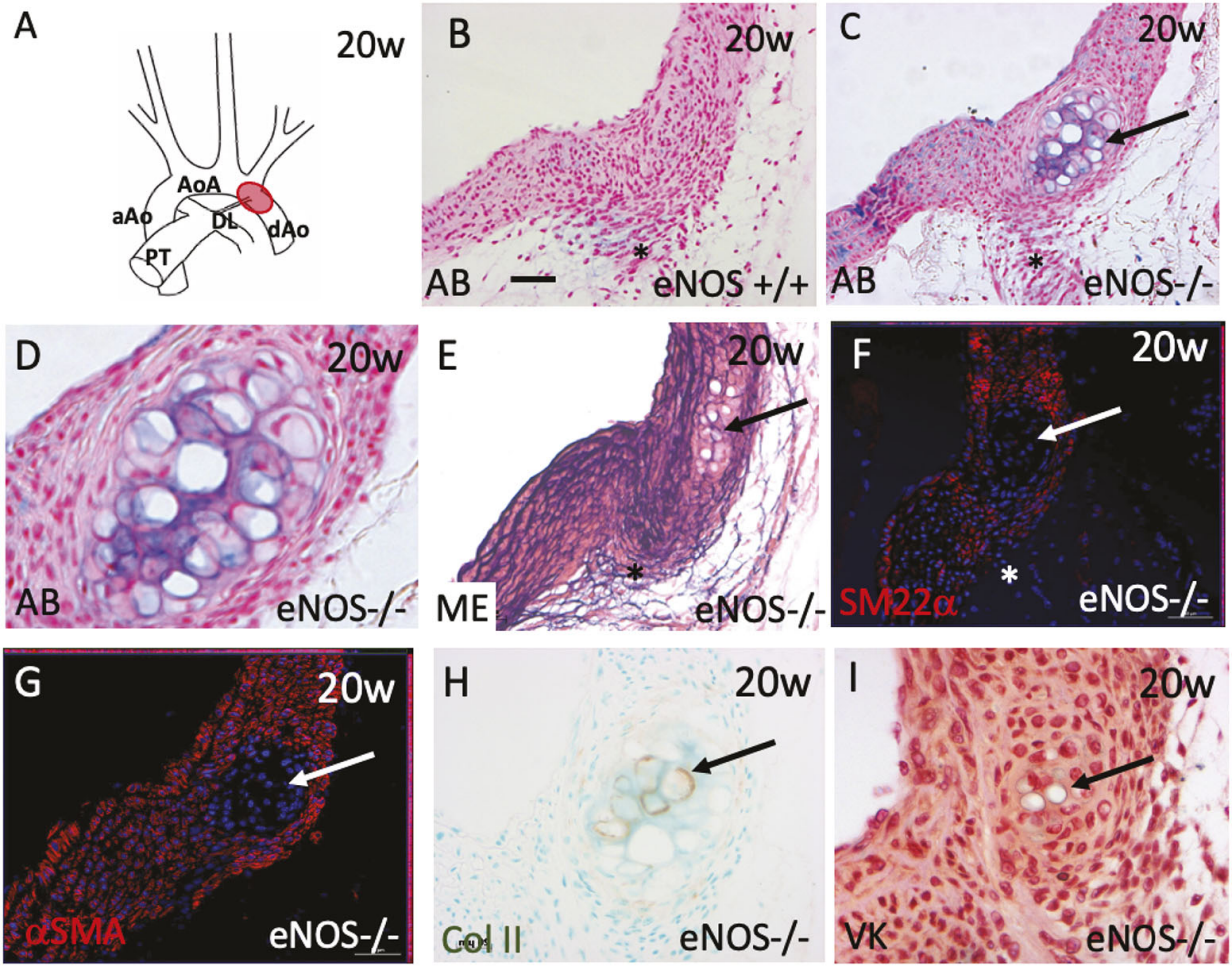
**Cartilaginous metaplasia in the peri-ductal region in eNOS null adults.** (A) Cartoon showing the thoracic aortic tree. The peri-ductal region is marked in red. (B-D) Alcian Blue staining reveals that nodules of vacuolated cells are found in the periductal region of eNOS nulls (arrow in C) but not wild-type controls (B) at 20 weeks (*n*=26). D is a higher magnification image of the nodule shown in C. (E-G) Smooth muscle cell markers, including Miller's elastin (ME; E), SM22α (F) and αSMA (G), are deficient in the region of the vacuolated nodules in eNOS nulls (*n*=3 for each marker). (H,I) The nodules are immunolabelled using antibodies raised against collagen II, suggesting that they are cartilage (H), but are only minimally labelled by Von Kossa staining (I), showing that they are not ossified (*n*=3 for each marker). AB, Alcian Blue; aAo, ascending aorta; AoA, aortic arch; dAO, descending aorta; ME, Miller's elastin; PT, pulmonary trunk; VK, Von Kossa. Arrows indicate the cartilage nodules. Asterisks indicate the ductal ligament. Scale bar: 50 µm in B,C,E-G; 125 µm in D; 80 µm in H,I.

Although Alcian Blue did not reveal changes in the amount of extracellular matrix, it unexpectedly revealed foci of intense staining in the post-ductal region in all of the 20-week-old eNOS null mice examined (*n*=26), but in none of the controls (Fig. 3A-D). These nodules were composed of vacuolated cells reminiscent of chondroblasts or osteoblasts and only became apparent at the time of sexual maturity, as smaller nodules were present at 10 weeks of age but not in juvenile (P21) or neonatal (P1-P2) animals (Fig. S4A-D).

In contrast to surrounding medial smooth muscle cells, the vacuolated cells could not be labelled for Miller's elastin or with a range of smooth muscle-specific reagents, including antibodies to SM22α and αSMA (Fig. 3E-G). However, the cells did label with collagen II antibody (Fig. 3H). Von Kossa staining for bone showed only minimal staining in the nodules (Fig. 3I). Thus, on the basis of the Alcian Blue and collagen II labelling, we concluded that these nodules represented aberrant cartilage formation within the aortic media. This type of pathology is known as cartilaginous (chondrous or chondroid) metaplasia (Hadjiisky et al., 1979). The localised peri-ductal distribution of the eNOS chondroid dysplasia suggested the possibility of a relationship with specific progenitor cell types, as the aorta in this area has contributions from different progenitor populations, notably neural crest cells (NCCs) and somitic mesoderm (Waldo et al., 2005; Wasteson et al., 2008; Choudhary et al., 2007; Harmon and Nakano, 2013).

### Disturbed cell and lineage boundaries in the peri-ductal aorta of eNOS null mutants

**Fig. 4.**
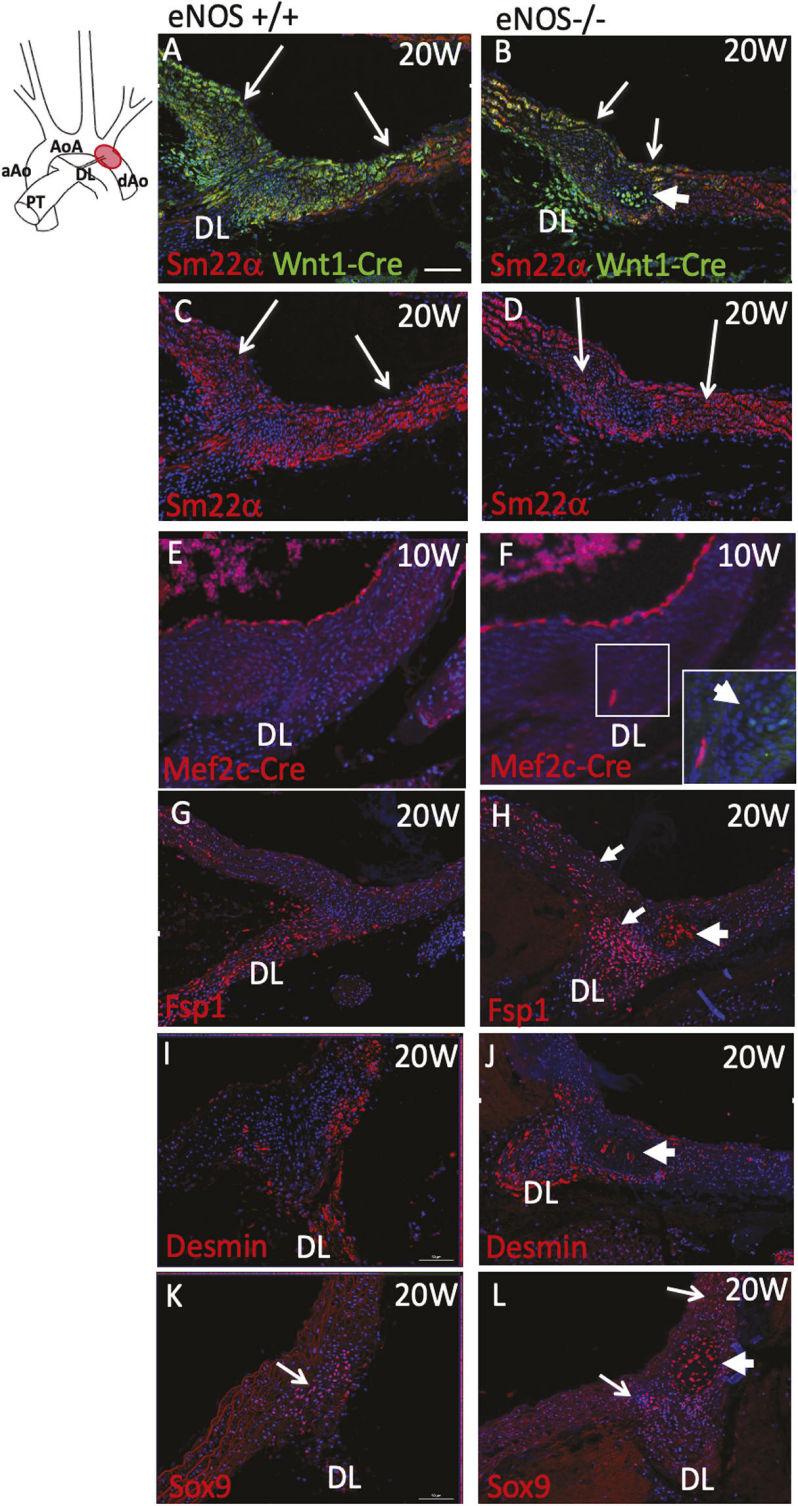
**Disrupted lineage boundaries and exacerbated ductal markers in the periductal region of the aortic arch in adult eNOS null mice.** A cartoon of the region of interest is shown at the top of the figure. *n*≥3 for each genotype and marker. (A-D) Frontal images through the thoracic aortic tree showing the distribution of Wnt1-Cre-labelled NCCs (green) in the peri-ductal region from controls and eNOS nulls at 20 weeks. Whereas NCCs extend throughout the periductal region in wild-type aorta (thin arrows in A), the periductal region is markedly deficient in NCCs in the eNOS^−/−^ (thin arrows in B). However, the cartilaginous metaplastic nodule found in eNOS null animals, which occurs immediately distal to the attachment of the ductal ligament in the NCC-deficient region, is packed with NCC-derived cells (thick arrow in B). SM22α staining (C,D) of the same sections in A,B shows that staining is reduced in the periductal region in wild types and eNOS nulls (arrows), with a similar pattern in both. (E,F) Mef2c-AHF-Cre-labelled SHF-derived cells in the endocardium appear similar in controls and nulls, with no labelling of SHF-derived cells in the cartilaginous nodules (thick arrow in F). A high magnification of the nodule is shown in the bottom left of F. (G-L) Fsp1 (G) and desmin (I) are abundant in the ductal ligament but not in the adjacent aortic media in control aorta; Fsp1 is expanded in the periductal region (thin arrows in H,J) and both are also found in the cartilaginous nodule in the eNOS nulls (thick arrows in H,J). Sox9 is restricted to the periductal region in control animals (arrow in K). This region, which includes the cartilaginous nodule (thick arrow in L), is expanded in the eNOS nulls (thin arrows in L). DL, ductal ligament; Mef2c-Cre, Mef2c-AHF-Cre; W, weeks. Scale bar: 50 µm.

To determine whether the cartilage nodules arose from NCCs, the eNOS null mice were inter-crossed with Wnt1-Cre and fluorescent reporter mouse lines. In normal control animals at 20 weeks of age, NCC-derived cells were found throughout the media of the aortic arch, extending beyond the position of the ductal ligament, which was also derived from NCC (Fig. 4A). The eNOS null animals revealed a disturbed NCC distribution. Although the ductal ligament retained abundant NCC, there was a striking reduction in Wnt1Cre-labelling within the periductal region of the aortic arch (Fig. 4B). The cartilage nodules all lay within the abnormally NCC-depleted territory, but their cells were labelled by a Wnt1-Cre lineage marker, indicating that they were derived from NCCs (thick arrow in Fig. 4B). SM22α staining was generally reduced in the periductal region compared with the surrounding aortic media but was comparable between controls and eNOS null mutants (Fig. 4C,D). To identify any ectopic cells of second heart field origin (normally restricted to the ascending aorta), similar experiments were carried out by inter-crossing the eNOS line with Mef2c-AHF-Cre mice. As expected, Mef2c-AHF-Cre labelled cells were found in the endothelium of the vessels but were not found in the cartilage nodules (Fig. 4E,F).

As the tunica media of the ductus arteriosus develops from NCCs and after its closure remains as a ligamentous remnant, we asked whether the cells within the isolated nodules of cartilaginous metaplasia shared phenotypic characteristics with ductal ligament cells. Fibroblast specific protein 1 (Fsp1) (Strutz et al., 1995) is normally expressed in the ductal ligament but not in the surrounding smooth muscle cells. In the eNOS null mice, its distribution was expanded to the SM22α-depleted peri-ductal region and was also present in the cells of the cartilaginous metaplasia nodules (Fig. 4G,H). Desmin, an intermediate filament protein that is normally expressed in the ductus arteriosus and later in the ductal ligament (Bergwerff et al., 1999), was found in the ductal ligament with some expression in vascular smooth muscle cells (SMCs) in the abluminal tunica media, although not in the ad-luminal or mid-luminal parts. In the eNOS null mice, the ad-luminal and mid-luminal parts remained devoid of desmin expression but there were a few desmin-positive cells in the nodules (Fig. 4I,J). Finally, *Sox9*, a gene that is expressed by the progenitors of tendons, ligaments and cartilage (Sugimoto et al., 2013; Liu et al., 2017), was found in the media in this region, in continuity with *Sox9*-expressing cells in the ductal ligament. Expression of *Sox9* was expanded in the eNOS null mutant mice to include the nodules (Fig. 4K,L).

**Fig. 5.**
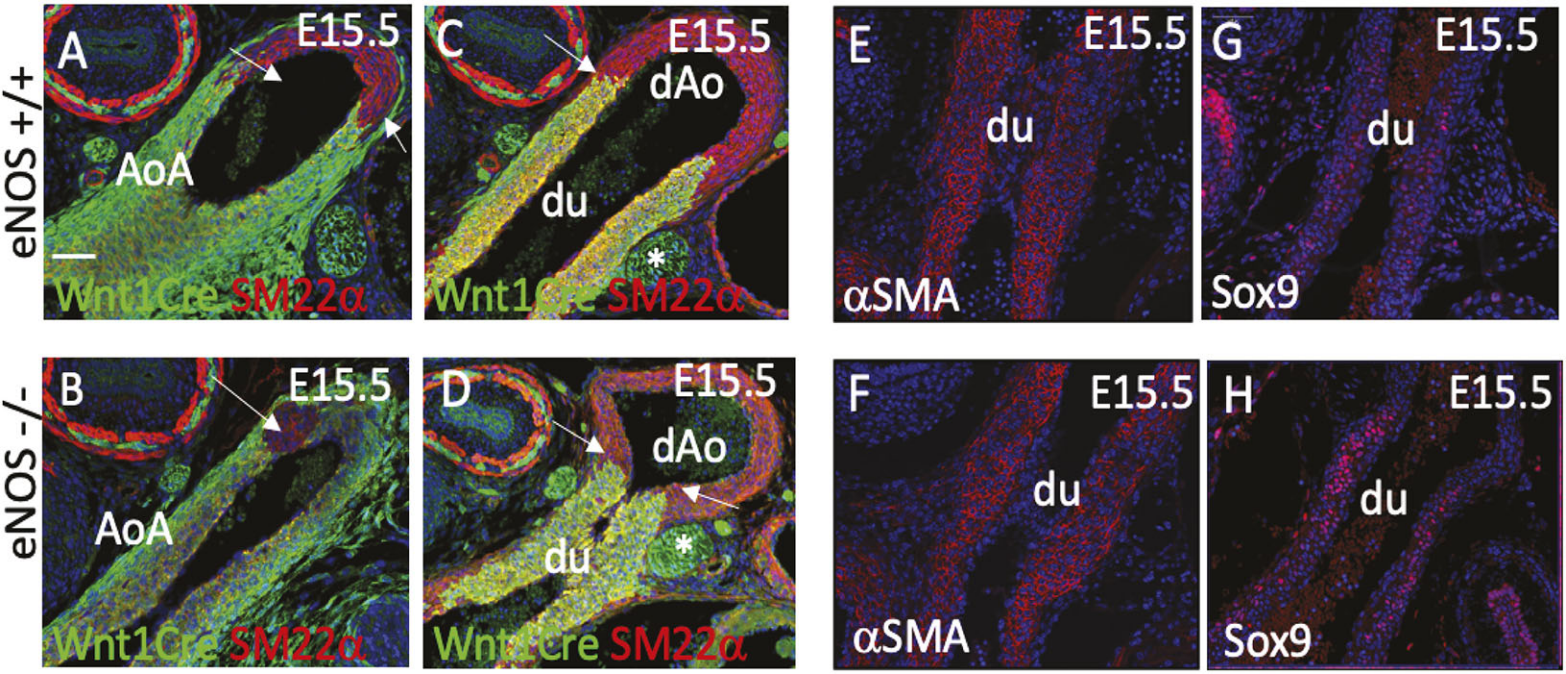
**Disruption of NCC differentiation in eNOS null fetuses.** (A-D) Overall patterns of NCCs (Wnt1-Cre) in the aortic arch and ductus arteriosus are similar in the control and eNOS nulls at E15.5. (E,F) Immunolabelling of αSMA at E15.5 shows similar expression in the ductus arteriosus of eNOS null fetuses and control littermates. (G,H) Sox9 is increased in the ductus arteriosus of eNOS nulls compared with control littermates at E15.5. *n*≤3 for each genotype for each marker. AoA, aortic arch; dAo, descending aorta; du, ductus arteriosus. Arrows in A-D indicate the boundary between NCC-rich and depleted areas. Scale bar: 50 µm.

As the abnormalities in the great arteries in eNOS mutants were first observed shortly before birth, we asked whether defects in the distribution of NCCs we observed with the Wnt1-Cre lineage tracing were apparent in the periductal region at this early stage. As in wild-type animals, NCCs were found throughout the aortic arch and ductus arteriosus at E15.5, but there was evidence of a loss of NCCs from the aortic arch by P1 (Fig. 5A-D; Fig. S5A-J). Although the expression of SMC markers in the aortic arch and ductus arteriosus were comparable between controls and eNOS mutants, there was greater staining for Sox9 and desmin in the mutant ductus arteriosus compared with controls (Fig. 5E-H; Fig. S5K-N).

Taken together, these studies show that the NCC-derived cells within nodules of cartilaginous metaplasia share phenotypic features with cells of the ductal ligament. Moreover, this region is already abnormal in eNOS null mutants from late gestation, with aberrant expression of pro-chrondrogenic markers.

### Loss of NCC-derived neurons in the periductal region

**Fig. 6.**
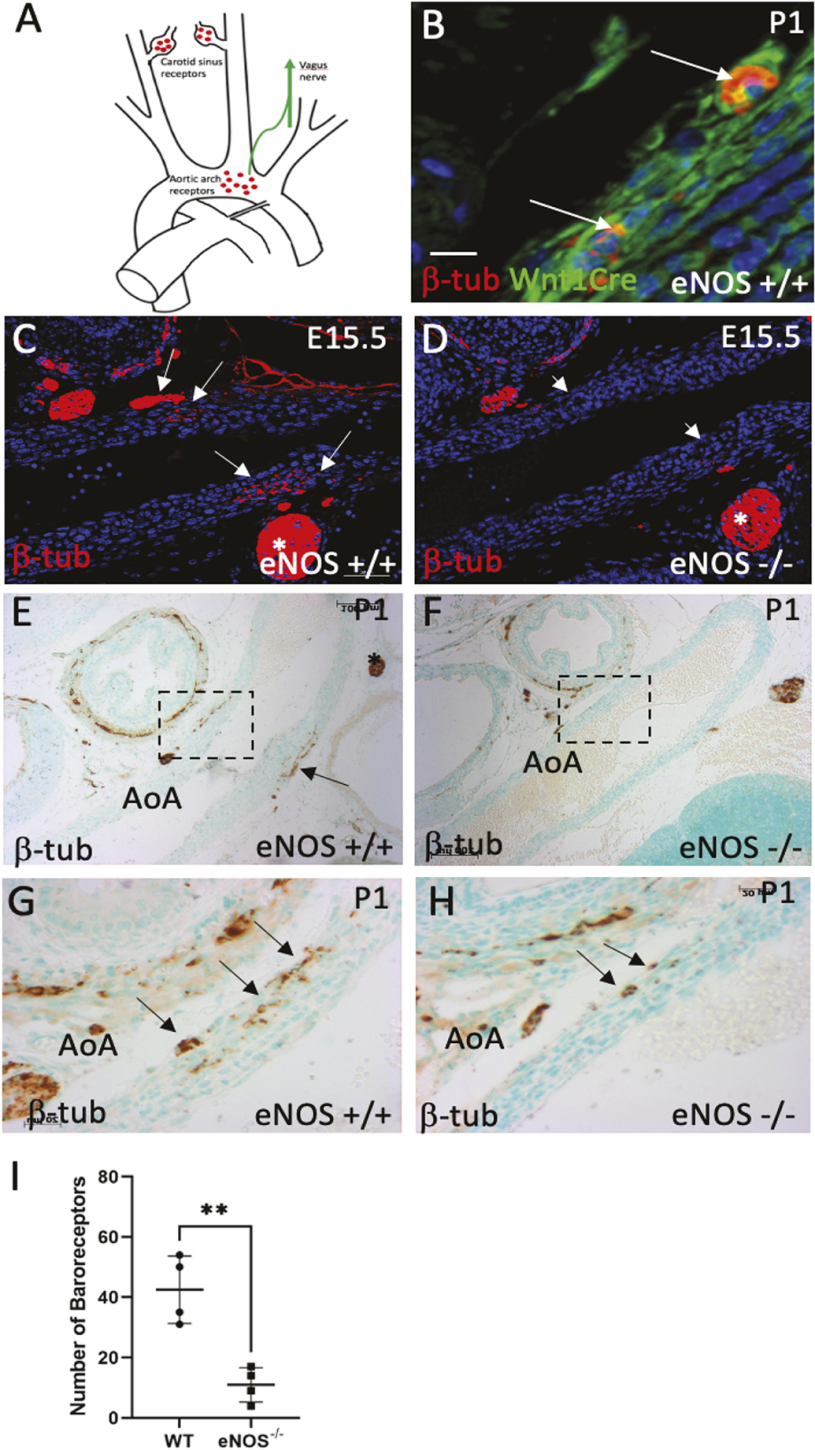
**Baroreceptors are disrupted in the aortic arch of eNOS nulls.** (A) Cartoon showing the position of baroreceptors in the aortic arch. (B) Nerve bodies in the aortic arch are NCC derived [co-labelled with β3 tubulin (β-tub; red) and Wnt-1Cre/eYFP (green)]. (C-H) Nerve bodies (arrows) are abundant in the outer media/adventitia of wild-type embryos at E15.5 (C) and P1 (E,G) but are markedly reduced in eNOS^−/−^ at the same stages (D,F,H). Asterisks mark the recurrent laryngeal branch of the vagus nerve. (I) Graph quantifying nerve bodies in the aortic arch from wild type and eNOS mutants at P1, showing a significant reduction in the nulls (**P<0.01). *n*≥3 for each genotype at E15.5 and *n*≥4 for each genotype at P1. Data are mean±s.d. AoA, aortic arch. Scale bars: 3 µm in B; 50 µm in C,D; 100 µm in E,F; 20 µm in G,H.

In addition to SMC within the media of the thoracic great arteries, NCCs also contribute to the peripheral nervous system (Fig. 6A,B). Thus, in view of the reduced NCC contribution to the periductal region, we wanted to determine whether this might extend to a reduction in the baroreceptors in the aortic arch and the periductal region. In control animals at E15.5 and P1, neuronal bodies identified by β3-tubulin expression was apparent as clusters of cell bodies within the adventitial walls and outer media of the aortic isthmus, both proximal and distal to the joining of the ductus arteriosus (Fig. 6C,E,G). In eNOS null mice there was a significant reduction (confirmed by quantification) in neurons detected using β3-tubulin staining within the periductal arterial media, specifically in the area where the chondrogenic nodules form (Fig. 6D,F,H,I). Thus, loss of NCC in the periductal region leads to a reduction in the numbers of baroreceptors in the periductal region.

### Dynamic expression of eNOS at the cellular and subcellular level correlates with pathology localisation in the aortic tree

**Fig. 7.**
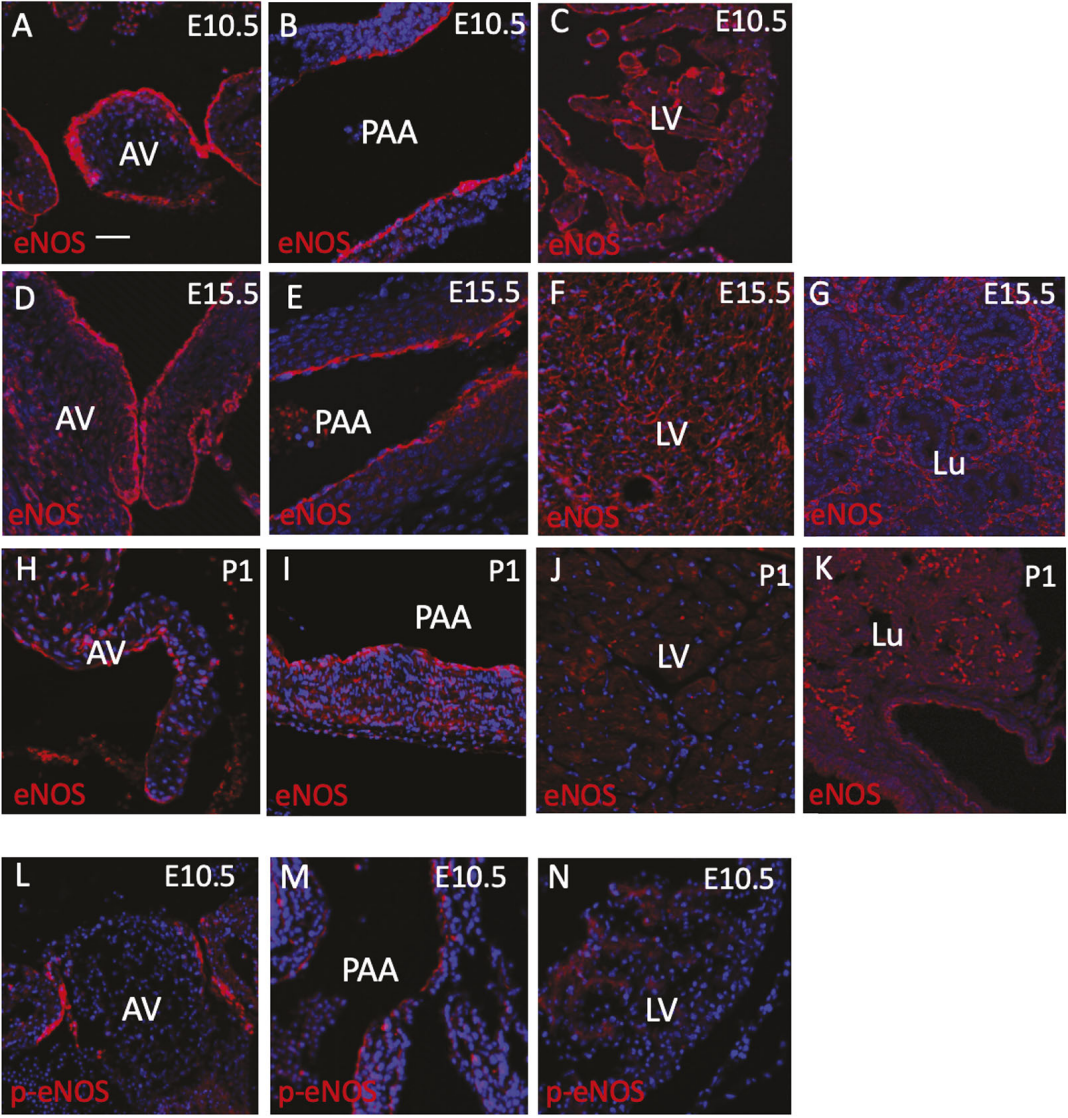
**eNOS expression during development of the cardiovascular system.** (A-K) At E10.5 (A-C), E15.5 (E-G) and P1 (H-K), eNOS protein is found in the endocardium of the heart valves (A,D,H), in the endothelium of the developing pharyngeal arch arteries (B,E,I) and in the endocardium of the ventricles (C,F,J), although decreasing in intensity as gestation proceeds. It is also found in the lungs at E15.5 and P1 (G,K). (L-N) p-eNOS-1177 (activated eNOS) is also expressed in the heart valves, pharyngeal arch arteries and, at lower levels, in the ventricles at E10.5. *n*=3 for each stage. AV, atrioventricular valve; Lu, lung; LV, left ventricle; PAA, pharyngeal arch artery. Scale bar: 50 µm in A,C,F,G,K,L,N; 100 µm in B,M; 75 µm in D,E,H-J.

To understand the origin of these diverse malformations, we examined the expression of eNOS in the affected structures during cardiovascular development and in the mature animal. At key developmental stages, eNOS was found outside the endocardium in regions that are associated with congenital malformation in the eNOS null mutants. At E10.5-E11.5, in addition to eNOS immunoreactivity being detected throughout the endothelium and endocardium, labelling in the developing ventricular myocardium and the media of the developing pharyngeal arch arteries was also found, although at lower intensity (Fig. 7A-C; Fig. S6A-C). Although the expression of eNOS in the endothelium and endocardium was maintained, by mid-late fetal development (E13.5-E15.5) the expression in the myocardium and tunica media had diminished, except in the SMCs of aortic arch and the ductus arteriosus (Fig. 7D-F; Fig. S6D-F,J,K). At E15.5, expression could also be seen in the developing lungs (Fig. 7G). At postnatal stages P1 and P21, the expression in the heart was limited to the endocardium covering the cardiac valves and the endothelium of the blood vessels. eNOS expression was also found in the lungs at these stages (Fig. 7H-K; Fig. S6G-I).

Activation of eNOS occurs through phosphorylation of a serine residue positioned at 1176 in the peptide (Kolluru et al., 2010), We were able to identify this phosphorylated isoform of eNOS using a specific antibody (p-eNOS-S1177). Immunostaining with p-eNOS-S1176 was found at the highest levels in the cushion/valve endocardium throughout development and postnatally. It was also observed at low levels in the endothelium of the developing pharyngeal arch arteries and appeared to be absent from the ventricles at E10.5, E15.5 and beyond (Fig. 7L-N; Fig. S6L-O).

### Notch signalling is disrupted in the developing PAA in eNOS null mutants

The cartilaginous metaplasia we see in adult eNOS null animals is similar to that described when Jag1 was deleted from SMCs (Briot et al., 2014), although in these cases there was no regional restriction. We could not detect Notch1 or Jag1 signalling in the thoracic aorta in the adult. However, although the cartilaginous metaplasia we see in eNOS nulls does not appear until early adulthood, we first saw abnormalities in the arch arteries during late fetal life. We therefore asked whether they might arise by a similar mechanism, from abnormal SMC differentiation, potentially related to disrupted Notch signalling in the fetal period.

**Fig. 8.**
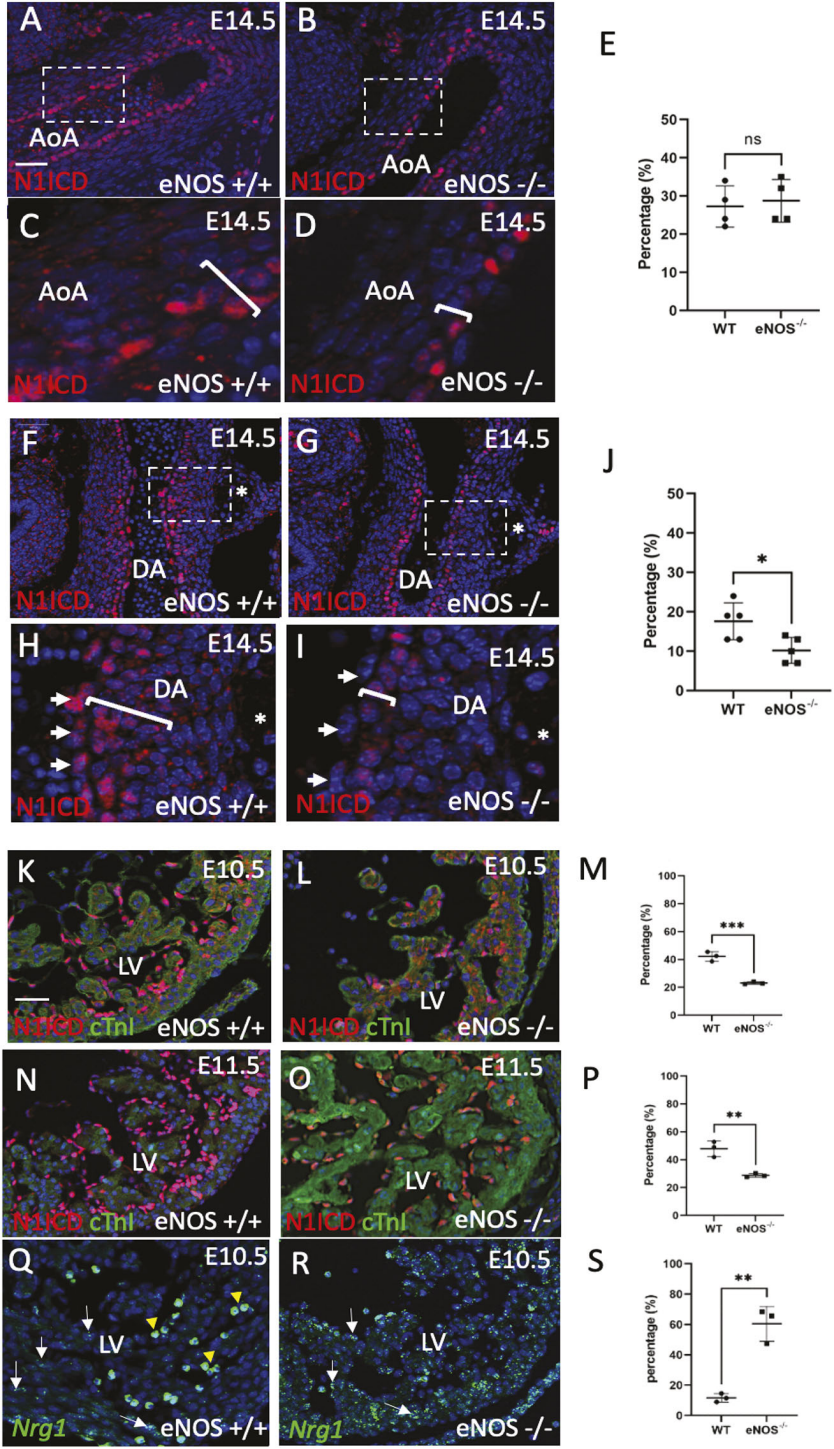
**Disrupted signalling in the developing ductus arteriosus and ventricles.** (A-J) The outlined areas in A,B,F,G are enlarged in C,D,H,I. (A,C,F,H) Notch1ICD is expressed in the innermost (luminal layers) of SMC in the media of the aortic arch (white brackets) as well as the ductus arteriosus in wild-type E14.5 fetuses. (H) It is also expressed in the endothelium (arrows) and in three or four layers of SMC (white bracket) of the ductus arteriosus. Notch1ICD immunoreactivity is only found in the most inner layer of SMC in the aortic arch and ductal media in eNOS null fetuses (B,D,G and bracket in I) and is lost in the endothelium of the ductus arteriosus (arrowheads in I). (E,J) Quantification of the N1ICD-positive cells in the aortic arch (E; *n*=4 for each genotype) and ductus arteriosus (J; *n*=5 for each genotype) showing that there is a significant difference between the percentage of N1ICD-positive cells in the ductus arteriosus of wild types, compared with eNOS nulls (*P<0.05). Data are mean±s.d. (K-S) Notch1ICD appears reduced in the ventricles of eNOS nulls compared with wild-type controls at E10.5 and E11.5 (K,L,N,O; *n*=3 for each genotype at both stages). In contrast, *Nrg1* mRNA appears more abundant in the ventricular endocardium of eNOS nulls at E10.5 than it is in wild-type littermates (Q,R; *n*=3 for each genotype). (M,P,S) Graphs showing quantification of Notch1 ICD (M,P) and *Nrg1* (S) positive cells in the ventricles, confirming that Notch1ICD is reduced and *Nrg1* increased in the ventricles of eNOS nulls compared with wild types. **P<0.01, ***P<0.001. Data are mean±s.d. AoA, aortic arch; DA, ductus arteriosus; LV, left ventricle. Scale bars: 50 µm in A,B,F,G; 200 µm in C,D,H,I; 50 µm in K,L,N,O,Q,R.

We looked at the expression patterns of the Notch 1 intracellular domain (N1ICD) and Jag1 in eNOS null and control embryos when SMC differentiation is occurring in the PAA. In control embryos at E14.5, N1ICD, which delineates active Notch1 signalling in the nucleus, was expressed in the SMC closest to the endocardium throughout the thoracic aortic tree, including the aortic arch, ductus arteriosus and descending aorta (Fig. 8A-I); it was also found in the endocardium restricted to the ductus arteriosus (arrowheads in Fig. 8H). Notch1ICD was absent from the endothelial cells of the ductus arteriosus of eNOS null mice at E14.5 (arrowheads in Fig. 8I; see also Fig. S7) and was also decreased in the media of the ductus arteriosus compared with controls (Fig. 8F-J). Jag1 protein was found in the membrane of cells throughout the wall of the aortic arch, including the region where the ductus arteriosus joins the arch (Fig. S7) but was more restricted in the ductus arteriosus, with the SMC closest to the intima having the highest levels (Fig. S7). Jag1 appeared downregulated in the inner layers of the media of the ductus arteriosus in some eNOS mutants when compared with control littermates (Fig. S7), although this was variable. There were no reproducible differences in Dll4 between control and eNOS mutants at this time point (Fig. S7).

These data thus show altered Notch signalling in eNOS null fetuses that precedes altered pro-chondrogenic signalling. This signalling occurs during a period of development that is crucial for differentiation of progenitors into the SMCs that contribute to the aortic arch in adulthood.

### Notch signalling is disrupted in eNOS null heart during development

Notch pathway mutants have abnormalities in the development of the ventricular myocardium that superficially resemble those we see in eNOS null mutants at E10.5 (Grego-Bessa et al., 2007; D'Amato et al., 2016). Moreover, Notch1 signalling has been shown to play an antagonistic role with neuregulin (Nrg1) to regulate the architecture of the ventricular trabeculae (del Monte-Nieto et al., 2018).

We looked at the distribution of Notch1ICD and Jag1 at E10.5 in eNOS null embryos and controls. Whereas Notch1ICD was abundant at the base of the forming trabeculae of control embryos at E10.5, it was less apparent in the eNOS null mutants (Fig. 8K-M). Similarly, at E11.5 there was reduced Notch1ICD expression in the ventricular endocardium of eNOS null embryos compared with wild-type littermates (Fig. 8N-P). In contrast, Jag1 was expressed only at a low level in the chamber myocardium in control and eNOS null mutants at E10.5 and E11.5 (Fig. S8). However, *Nrg1* expression levels, as detected by RNAScope, were significantly increased in the ventricles of eNOS null embryos at E10.5, compared with wild-type embryos (Fig. 8Q-S). There were no reproducible differences in *Vegfa, Has2* or versican expression between controls and mutants (Fig. S8). Thus, Notch1 and *Nrg1* were disrupted in the developing ventricles, before overt abnormalities in trabeculae development are observed.

## DISCUSSION

This study indicates the range of essential roles performed by eNOS in cardiovascular and respiratory development from early gestation to adulthood. Importantly we show that defects in myocardial development led to early embryonic lethality, with added high risk of death in the immediate neonatal period, likely due to the combination of abnormal cardiac and pulmonary development. However, this 25% mortality in eNOS null pups is much lower than the previously reported 85% (Feng et al., 2002) and may relate to the background effect of different strains of C57Bl/6 mice used in the studies, as well as to the extensive outcrossing we employed to ensure vigour in our colony: to obtain sufficient eNOS pups to maintain the line, we found it necessary to outcross the mice every second generation. We show that the predominant defects in eNOS mutants are found in the ventricular myocardium, with a thinned compact wall and prominent, retained trabeculae; these defects can lead to postnatal death related to heart failure. The origin of these defects arises early in embryogenesis and may also be the cause of death *in utero*. Major abnormalities in the thoracic aorta and its associated aortic arch arteries were also found in eNOS null pups. These arise late in gestation and result in structural arch artery anomalies, including right-sided aortic arch and retro-oesophageal subclavian artery, which result from abnormal regression of the left and right 4th pharyngeal arch artery, respectively (Levitt and Richter, 2007), and also persistent ductus arteriosus. Importantly, they are also associated with predisposition to aortopathy and complete penetrance of cartilaginous metaplasia, which is restricted to the periductal region. Thus, all of these abnormalities in the arterial tree map to the 4th and 6th PAA. Similarities in the defects observed, and evidence of abnormal signalling in the eNOS null embryos, suggest that eNOS signals via the Notch pathway to regulate myocardial and SMC differentiation and maturation. Moreover, increases in *Nrg1* expression in the ventricular myocardium of eNOS mutant embryos, suggest that the interplay between eNOS, Notch1 and Nrg1 may be crucial for normal development of the trabeculae. We suggest that, as well as regulating vascular tone, eNOS is playing crucial roles in development and maturation of the ventricular myocardium and the media of the aortic vasculature. These result in clinically relevant structural heart defects and cardiovascular disease that are distinct from its effects on endothelial dysfunction.

The best described pathologies in eNOS null mice – BAV and hypertension – are seen only in the healthiest fraction of eNOS null mutants, as the rest of the mutants die either before or around the time of birth, principally from heart failure. Thus, the adult phenotype is the mildest end of the phenotypic spectrum and although some of these pathologies may reflect primary roles for eNOS in the mature vasculature, some may be secondary to congenital abnormalities. For example, the large VSD and abnormal ventricular myocardium we see in some adults was almost certainly present before birth, as is seen in the majority of eNOS null fetuses. Other disease states, such as hypertension, may also be affected by developmental defects. eNOS null mice are hypertensive, and high-pressure baroreceptors that sense blood pressure and feed back to modulate vasal tone are localised within the adventitia of the aortic arch in both the fetus and the adult. These baroreceptors are stretch receptors that sense distension of the arterial wall in response to increased blood pressure (reviewed by Reutersberg et al., 2022). Studies in which eNOS was reconstituted in the endothelium of adult eNOS null mice showed that although this restored vascular reactivity, the mice continued to be hypertensive (Suvorava et al., 2015). Although unexplained in the original publication, it was suggested it might result from extra-endothelial roles for eNOS. However, our data suggest that this may, at least in part, be the consequence of the absence and/or reduction of NCC-derived baroreceptors in the aortic arch of eNOS null mutants. Conversely, a decrease in baroreceptor activation causes heart rate to increase and blood pressure to increase. This negative-feedback loop causes heart rate to drop and blood pressure to decrease. Thus, in their absence, there is limited capacity to respond to normalised endothelial function and thus blood pressure remains high. As well as having relevance to eNOS mutants, this may also be relevant to patients with repaired co-arctation of the aorta, who frequently remain hypertensive despite surgical correction of the lesion (reviewed by Kenny and Hijazi, 2011). Pre-ductal co-arctation of the aorta affects the isthmus of the aortic arch, which is a NCC-derived segment. It seems likely then that the NCC-derived baroreceptors in the aortic media/adventitia are deficient in these patients, and thus the vasculature cannot respond effectively despite the cause of the increased blood pressure being removed. It has also been suggested that the aorta close to the narrowed segment shows decreased contractility, caused by reduced SMC content and increased collagen, that alters distensibility of the vessel and thus produces an altered baroreceptor response (Sehested et al., 1982; Kenny and Hijazi, 2011). This situation is strikingly similar to the abnormal expression of proteins in the periductal region of eNOS null mice.

Cartilaginous metaplasia, located specifically in the peri-ductal region of the aortic arch, was one of the most striking and fully penetrant phenotypes we observed in adult eNOS null mice. The specific and highly reproducible location of these cartilage nodules raises an interesting question about their origin. Although this phenotype was only ever seen after weaning, we could detect excessive Sox9 expression in the ductus arteriosus of eNOS nulls in late gestation, suggesting that, although this pathology is not seen until much later, it might have an origin in fetal life. The periductal region is a nexus, with cells from different embryonic precursor structures (4th and 6th PAA, and dorsal aortae) and different embryonic origins [endothelium (e.g. primitive vascular plexus and secondary heart field) and SMC (e.g. NCC and somites)] forming seamless anatomical, but long-term lineage and molecular, boundaries. Thus, cells of different embryonic origins border each other in this region (Topouzis and Majesky, 1996), although whether the differences in origin of the mature endothelial cells and SMC is relevant in the mature organism remains unclear (Pfaltzgraff et al., 2014). Irrespective of this, the loss of NCCs from the periductal region of the aortic arch in the eNOS null embryos during late gestation is associated with a shortening of the aortic isthmus that corresponds to the 4th PAA-derived segment. It thus seems likely that eNOS is playing a distinct role in the arterial wall of the 4th (aortic arch) and 6th (ductus arteriosus) PAAs, either within the secondary heart field-derived endothelium or the NCC-SMC, that is different from that in the adjoining vascular plexus-derived endothelium and somite-derived SMCs of the descending aorta. This is supported by the observation that, as well as localising to the endocardium, eNOS is expressed (although at only low levels) in the developing arterial media of the aortic arch and ductus arteriosus. Similarly, eNOS is expressed in the ventricular myocardium at E10.5-E11.5. Thus, the developmental defects seen in eNOS null mice correlate with extra-endocardial expression of eNOS.

The observation of ectopic expression of the ductus arteriosus markers Sox9, Fsp1 and desmin in the cartilage nodules in the peri-ductal region of the aorta, together with their overexpression in late gestation, suggests that these might be causally related. Sox9 is a key transcriptional regulator of chondrogenesis and has to be actively suppressed in the developing aortic media. During gestation, Notch signalling, via Jag1, promotes vascular SMC differentiation and inhibits Sox9 expression, thus blocking chondrogenesis (Briot et al., 2014). Interestingly, Feng et al. (2010) showed that defects in SMC differentiation in mutants where Jag1 was deleted in all SMC (using SM22α-Cre) were restricted to the ductus arteriosus and periductal aortic arch, as are the defects we see in the eNOS null mutants, suggesting a unique and essential role in these regions. Thus, the pathological mechanism may involve Notch signalling, which has been shown previously to be important for the differentiation and maturation of different SMC lineages (High et al., 2007, 2008, 2009; Manderfield et al., 2012). We show that in late gestation wild-type embryos, there are marked and reproducible differences in the localisation of Notch1ICD between the endothelium and SMC of the aortic arch and/or ductus arteriosus and those in the descending aorta, suggesting that these patterns may be related to the lineage of origin of the cells, as has been previously reported for Notch3 (Granata et al., 2015). These patterns are altered in eNOS null fetuses in mid-gestation, before the changes of PAA structure and marker expression are apparent, with loss of Notch1ICD from the endothelium of the ductus arteriosus and from the SMC closest to the intima of the ductus arteriosus and the isthmus of the aorta. As Notch- and Jag1-dependent signalling has been proposed to act as a lateral induction mechanism to induce differentiation of SMCs from the intima first, then through the entirety of the arterial wall (Manderfield et al., 2012), the disruption of this pathway in the eNOS nulls could account for the abnormalities seen in the walls of the aortic arch and ductus arteriosus. Thus, eNOS may act in a pathway with Notch1 and/or Jag1 in the developing pharyngeal arches, as has been suggested in other tissues (Chang et al., 2011; Patenaude et al., 2014). In support of this idea, Notch1^+/−^;eNOS^−/−^ developed more severe aortopathy than did Notch1 heterozygotes or eNOS null animals (Koenig et al., 2015).

Notch signalling is also important for the growth of the ventricular myocardium (reviewed by Grego-Bessa et al., 2007; D'Amato et al., 2016). Disruption of Notch signalling in the endocardium and in ventricular cardiomyocytes results in failure to form trabeculae early in gestation (via Notch1–Dll4 interactions), and failure to form a compact ventricular wall later in development (via Notch1–Jag interactions) (D'Amato et al., 2016). These latter defects resemble those we see in the eNOS null embryos, and indeed Notch1ICD is reduced in the developing eNOS^−/−^ ventricular myocardium at E10.5. There is evidence that eNOS and Notch can act within the same signalling pathway, with some studies suggesting that Notch acts upstream of eNOS to activate its production in tumour and valve endothelial cells (Chang et al., 2011; Patenaude et al., 2014). In contrast, endothelial eNOS can induce Notch expression in neighbouring cells in glioma cells in the perivascular niche (Charles et al., 2010). Thus, it is plausible that eNOS is regulating Notch signalling in a cell type-specific manner in the PAA endothelium and ventricular endocardium, and that disruption of this results in defects similar to those seen when Notch signalling is disrupted directly. Intriguingly, *Nrg1* expression was significantly increased in the ventricular myocardium of eNOS null embryos compared with controls. Nrg1 has been suggested to work together with Notch1 to regulate development of the ventricular trabeculae architecture, via their effects on the ECM and, in the case of Nrg1, on Vefga expression (del Monte-Nieto et al., 2018). Although Vegfa levels appeared unaffected in the eNOS null embryos, as did levels of the ECM molecule versican and the ECM synthesis gene *Has2*, the recent identification of Yap1 as a target of Nrg1 in the developing trabeculae (Grego-Bessa et al., 2023) suggests that this might be an interesting avenue for further study.

Variants of the *NOS3* gene are associated with CHD in human populations (Van Beynum et al., 2008; Kuehl et al., 2010; Zhou et al., 2014; Khatami et al., 2017). Our study, and those of others, support the idea that eNOS plays a crucial role during cardiovascular genesis and that variants in eNOS may be relevant to human disease. The observation that adult eNOS null mice that develop a range of cardiovascular diseases, including pulmonary arterial hypertension and cardiac dysfunction (Fagan et al., 2000; Li et al., 2004; Atochin and Huang, 2010), are relatively rare survivors that may carry some congenitally acquired malformations, strengthens the link between congenital and adult heart defects. It also suggests that the high levels of cardiovascular disease in children and adults with congenital heart disease (Brida and Gatzoulis, 2019; Wang et al., 2019) may, at least in part, be linked as primary abnormalities, rather than the cardiovascular disease developing as secondary sequalae from corrected or uncorrected malformations.

## MATERIALS AND METHODS

### Mouse strains

eNOS null mice were obtained from Jackson Labs (B6.129P2-Nos3^tm1Unc^/J; JAX 002684; Shesely et al., 1996) and genotyped as described previously. These were maintained on the C57Bl/6J background (Charles River Laboratories) either as a homozygous null line (eNOS^−/−^) or as a heterozygote cross (eNOS^+/−^ crosses), with, in each case, backcrossing to C57Bl/6J every second generation to maintain vigour within the line. Wnt1-Cre (Danielian et al., 1998) and Mef2c-AHF-Cre (Verzi et al., 2005) mice were inter-crossed with the eNOS null and the ROSA-Stop-eYFP (Srinivas et al., 2001) or mTmG (Muzumdar et al., 2007) reporter lines, and were used to follow cells of the relevant lineage/cell type. As both marker genes are targeted into the Rosa26 locus and there is no suggestion of differences in recombination in response to Cre between these two lines, the two lines were used interchangeably. Timed matings were carried out overnight and the presence of a copulation plug was designated embryonic day (E) 0.5. Littermate controls were used where possible. Where this was not possible, age-matched C57Bl/6J mice were used. Each experiment was carried out, at minimum, in triplicate.

Mice were maintained according to the Animals (Scientific Procedures) Act 1986, United Kingdom, under project licenses PPL 30/3876 and P9E095FF4. All experiments were approved by the Newcastle University Ethical Review Panel*.* For survival analyses, data was taken from eNOS^+/−^ inter-crosses before birth and eNOS^−/−^ inter-crosses, unless otherwise stated.

### Histological analysis

Harvested tissues were rinsed in ice-cold phosphate-buffered saline (PBS) and fixed in 4% paraformaldehyde at 4°C, the duration of which depended on the age of the embryo, before paraffin wax embedding. In the case of immunostaining for p-eNOS-S1177, the samples were fixed in 1% acetic acid in 95% ethanol at room temperature, then processed as for samples fixed in paraformaldehyde. Embryos were embedded in either a transverse, frontal or sagittal orientation. For each analysis carried out using embryos, a minimum of three embryos for each developmental stage and section orientation were used (biological replicates). Stage matching of embryos was carried out based on somite counting and other external features, such as limb maturity. Not all embryos/adult hearts were sectioned in their entirety, meaning that, for some, only the great arteries and valves were sectioned and analysed, with no analysis of the cardiac chambers. The analyses were carried out as separate experiments and a minimum of 5-10 sections (technical replicates) were analysed for consistency. For basic histological analysis, paraffin wax-embedded embryos were sectioned and stained with Haematoxylin and Eosin following standard protocols. For Alcian Blue staining of cartilage (Alcian Blue staining protocol), paraffin wax-embedded 8 µm sections were dewaxed and rehydrated then immersed in 15% w/v Alcian Blue (pH 0.75) for 10 min. They were then counterstained with Fast Red for 5 min, dehydrated and mounted before imaging. For Von Kossa staining, paraffin wax-embedded 8 µm sections were dewaxed, rehydrated and immersed in 1% silver nitrate, washed, then treated with sodium formamide. After a further washing step, the sections were placed in 5% sodium thiosulphate, counterstained with Fast Red, dehydrated and mounted before imaging using a Zeiss Axioplan microscope and Zeiss Axiovision software.

### Immunohistochemistry and immunofluorescence

Methodology for immunohistochemistry and immunofluorescence has been published previously (Phillips et al., 2013). Sections were cut from paraffin wax-embedded embryos at 8 µm using a rotary microtome (Leica). Slides were de-waxed with Histoclear (National Diagnostics) and rehydrated through a series of ethanol washes. For fluorescent immunohistochemistry, after washes in PBS, antigen retrieval was performed by boiling slides in citrate buffer (0.01 mol/l) at pH 6.3 for 5 min. Sections were blocked in 10% FCS and then incubated overnight at 4°C with the primary antibodies against the following proteins diluted 1/100 in 2% FCS: eNOS (Santa Cruz Biotechnology; sc-654), p-eNOS-S1176 (Sigma-Aldrich; SAB4504393), GFP (Abcam; ab13970), Sox9 (Abcam; ab185230), Collagen II (Abcam; ab3092), FSP1 (Millipore; S100A4), Desmin (Santa Cruz Biotechnology; sc-271677), β-tubulin (Sigma-Aldrich; T5293), alpha smooth muscle actin (Abcam; ab5228 and ab5694), SM22 alpha (Abcam; ab14106), cardiac Troponin I (HyTest; 4T21/2), Notch1ICD (Cell Signaling Technology; 4147), jagged 1 (Cell Signaling Technology; 2620), Dll4 (Santa Cruz Biotechnology; sc-28915) and versican (Merck, AB1032). After washing, sections were incubated at room temperature for 1 h, with secondary antibodies (diluted 1/200 in 2% FCS) conjugated to Alexa Fluor 488, Alexa Fluor 568 or Alexa Fluor 594 (Life Technologies, A11078, A11039, A11042 and A21203). Fluorescent slides were then mounted with Vectashield Mounting medium with DAPI (Vector Labs). For chromogenic immunohistochemistry and fluorescent Notch1ICD staining, endogenous peroxidases were inhibited by treating slides with 3% H_2_O_2_ for 5 min, then the slides were treated as if for immuno-fluorescence until the detection stages when biotin-labelled secondaries (diluted 1/200) were used, followed by an avidin-biotin complex conjugated to horseradish peroxidase (Dako) treatment. For chromogenic immunohistochemistry, diaminobenzidine (DAB; Sigma-Aldrich) staining was then used to visualise immunoreactivity. For fluorescent Notch1ICD staining immunoreactivity was observed using TSA Cyanine 3 System (Perkin Elmer, NEL704A001KT). TBST was used for all washes and dilution of antibodies instead of PBS. Each experiment was carried out in triplicate using separate embryos (at least three) and imaging multiple sections of the structure of interest where possible. Immunofluorescence images were collected with using a Zeiss Axioimager Z1 fluorescence microscope equipped with Zeiss Apotome 2. Acquired images were processed with AxioVision Rel 4.9 software.

### Elastin measurements

For measurements of overall elastin content, eNOS^−/−^ null mice aged P140 (*n*=6) and wild type mice at P140 (*n*=6) were examined. Histological sections of ascending aortic wall and aortic arch were imaged using an Axioplan Zeiss Brightfield Microscope (AxioVs40, V4.8.1.0) and were analysed using ImageJ 1.52p (Java 1.8.0_172). Quantification of elastin was made with respect to the aortic vessel wall. All the images were in 8-bit RGB format with identical resolution and magnification. Identification of elastin was carried out manually using the following RGB thresholds: hue (minimum=162, maximum=208), saturation (minimum=50, maximum=159), brightness (minimum=0, maximum=180). Total elastin content was expressed as a proportion of the volume of aortic vessel wall. The data were analysed with an unpaired two-tailed *t*-test using GraphPad prism 9 [v9.0.0(121)].

For quantification of the numbers of elastic laminae and elastin breaks, histological sections from the thoracic aorta and aortic arch were divided into two regions: the ascending aorta and aortic arch. Counts were performed at 40 µm intervals and averages for the whole region calculated. To avoid counting laminae that had passed out of plane as elastin breaks, adjacent sections were analysed when a break was suspected. A minimum of five animals were analysed for each genotype. The data were analysed with an unpaired two-tailed *t*-test using GraphPad prism 9 [v9.0.0(121)].

### Measurements of ventricular thickness and arch length

Wall thickness measurements were taken (using ImageJ) from three comparable sections, at four representative positions, in the ventricles (see Fig. S1H) from wild type and eNOS controls at E18.5 (*n*=5 for each genotype). At E10.5, the 4th and 6th pharyngeal arches were measured from images taken from ink injections (see below). Measurements at E15.5 and P1 were taken from transverse midline sections, normalised to the diameter of the oesophagus. Two measurements were taken of adult (20 week) arches, from frontal sections. These were between the brachiocephalic and left subclavian arteries, and between the left common carotid arch and left subclavian arteries. A minimum of *n*=5 per genotype were measured. The data were analysed with an unpaired two-tailed *t*-test using GraphPad prism 9 [v9.0.0(121)].

### Ink injections and analysis of the pharyngeal arch arteries

Analyses were carried out as described by Henderson et al. (2001). Briefly, E10.5 eNOS null and control embryos were dissected free of the yolk sac and the embryo was laid on its left side on top of the agar plate filled with PBS. The peritoneal membrane was removed to expose the heart. Closed forceps were used to steady the heart/outflow tract, then a pulled glass pipette needle, filled with India Ink (diluted 1:1 with distilled water), was inserted at the base of the outflow tract. The ink was gently expelled until the pharyngeal arch arteries could be visualised. The embryo was then imaged, before turning onto its right side to image the left pharyngeal arch arteries.

### Angiotensin II perfusion

These experiments were carried out according to Patel et al. (2015). Angiotensin II (Sigma-Aldrich) was dissolved in sterile water. This was loaded into Micro-osmotic pumps (model 1002; Alzet, Charles River Laboratories) with a pump rate of 0.25 µl/h so as to deliver a daily dose of 0.8 µg/g/day. Similar pumps were loaded with vehicle only. Twenty-week-old eNOS null mice were weighed and anaesthetised using isoflurane and carprofen. After an incision in the nape of the neck, the mini pumps (five mice with AngII, four mice with vehicle only) were inserted into a subcutaneous pocket formed between the skin and the muscle, and massaged gently into position. The wound was then closed and the mice were allowed to recover and were maintained for up to 4 days, before culling and dissection of the aortic tree. Notably, one out of three eNOS null animals perfused for 4 days suffered an aortic dissection and died before collection, so follow-up animals were perfused for only 3 days. These were processed for embedding and staining as for other samples.

### RNAscope

RNAscope was performed using the RNAscope Multiplex Fluorescent Reagent Kit v2 from Bio-techne (323100) according to the manufacturer's instructions. Briefly, embryos were fixed in 4% PFA at 4°C overnight, serially dehydrated using ethanol, and embedded in paraffin wax oriented for transverse sections. The embryos were then sectioned at 8 µm using a Leica microtome and sections were mounted on positively charged slides (Marienfield). The slides were then heated to 60°C for 10 min, allowed to cool to room temperature, and dewaxed in xylene for 6 min before dipping in 100% ethanol and air drying. After that, slides were incubated with hydrogen peroxide for 10 min at room temperature and rinsed in water before soaking in boiling target retrieval solution for 20 min using a steamer. After rinsing the slides in 100% ethanol and air drying, protease plus solution was added to the sections for 30 min at 40°C. RNA probes of channel 2 (Has2, 465171-C2) and channel 3 (Vegfa, 412261-C3) were diluted in a 1 in 50 volume of channel 1 probe (Nrg1, 418181) and incubated at 40°C for 2 h. All probes were from Bio-techne. After several sequential amplification and blocking steps, the slides were incubated in TSA Vivid Fluorophore kit (7527/1 KIT, 7523/1 KIT and 7526/1 KIT, Bio-techne) at a dilution of 1:1000 using TSA diluent. Finally, slides were mounted using prolong Gold antifade mounting (Thermo-fisher Scientific) and stored at −20°C until imaged.

### Quantification of baroreceptor numbers, Notch1-ICD and Nrg1 expression

Baroreceptors were visualised using an antibody raised against β-tubulin, visualised with DAB. Baroreceptors cell nuclei were counted over two matched sections covering the transverse aortic arch in wild-type and eNOS null mice at P1 (*n*=4 for each genotype).

The nuclei of cells expressing Notch1-ICD or Nrg1, as well as the total nuclei number (marked with DAPI), were counted in a representative 250×250 mm region of the left ventricular chamber [four sections from three controls and three eNOS null embryos at E10.5 (Nrg1 and NICD1) and E11.5 (NICD1)]. The marker-positive nuclei are presented as a percentage of total nuclei.

Notch1ICD nuclei numbers were counted from representative midline sections of E15.5 aortic arch (*n*=4) and ductus arteriosus (*n*=5), and were calculated as a percentage of the total number of cells in the arterial wall. Student's (unpaired two-tailed) *t*-test was performed using Graphpad prism to compare between values.

## Supplementary Material

10.1242/dmm.050265_sup1 Supplementary information
