# Supplementary material for: eNOS plays essential roles in the developing heart and aorta linked to disruption of Notch signalling
Source: Dis Model Mech. 2024 Jan 22;17(1):dmm050265. doi: 10.1242/dmm.050265 (PMC10846539; doi:10.1242/dmm.050265)
Supplement: Supplementary information [file dmm-17-050265-s1.pdf]

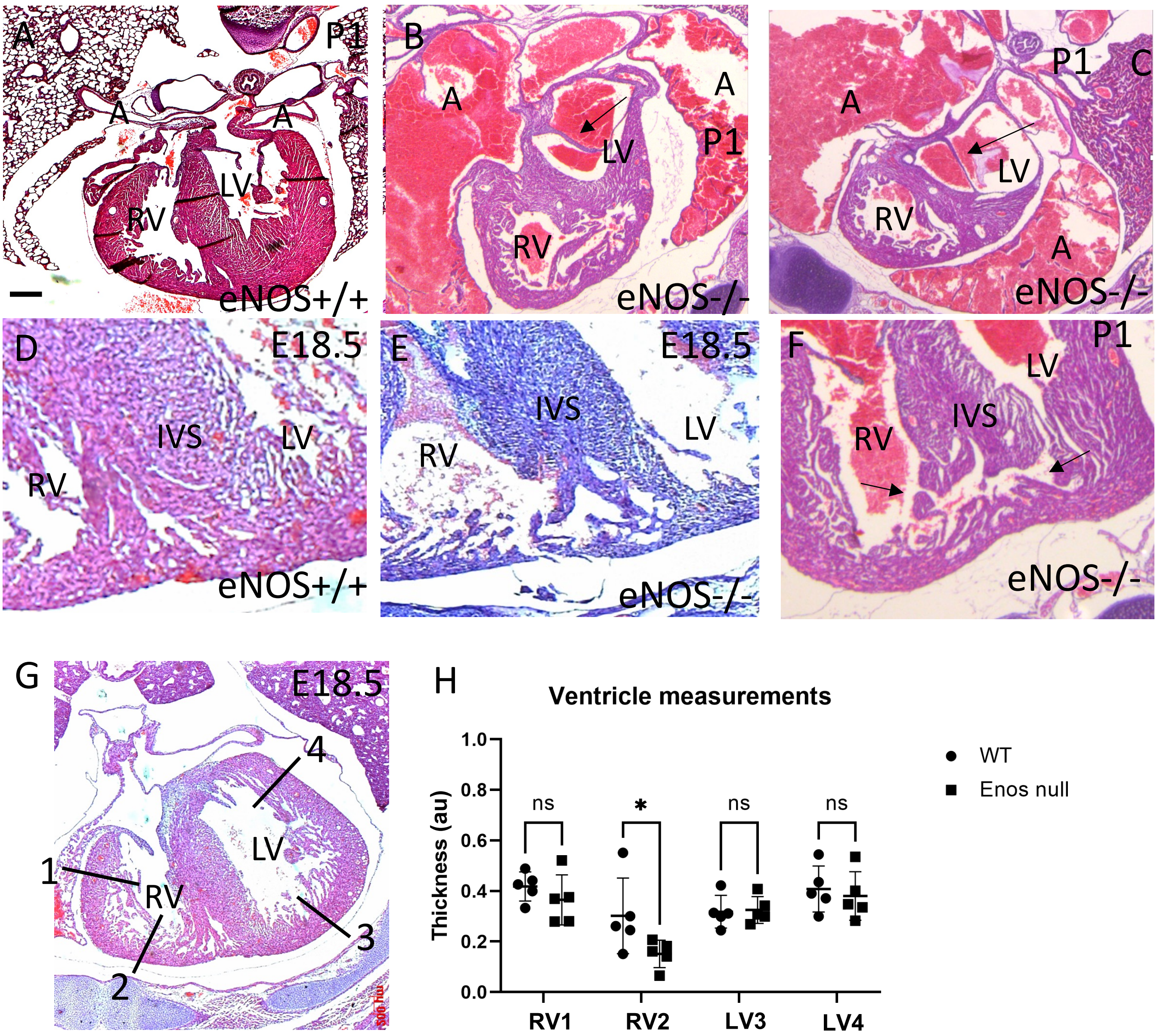

Fig. S1. Ventricular defects and wall measurements in eNOS mutants and controls. A-C) Grossly abnormal heart in an eNOS mutant at P1 (B,C), compared to a stage-matched wild type (A). The atrioventricular valves appear well formed (arrows) although the atria are grossly distended in the mutant. D-F) abnormal myocardium at the ventricular apex seen in an eNOS mutant at E18.5 (E; compare with wild type in D), although no mVSD can be seen. In contrast, a mVSD with clear communication between the ventricular chambers (arrows in F) can be seen in a mutant at P1. G) Illustration of position where ventricular measurements were taken. H) Measurements of ventricular wall thickness at E18.5 (n=5 for each genotype) show no differences between wild types and eNOS mutants except in the apical region of the right ventricle (au = arbitrary units).

A - atria, IVS = interventricular septum, LV = left ventricle, RV = right ventricle.

Scale bar in A-C,G= 440μm, D-F = 1200 μm. * denotes $P < 0.05$.

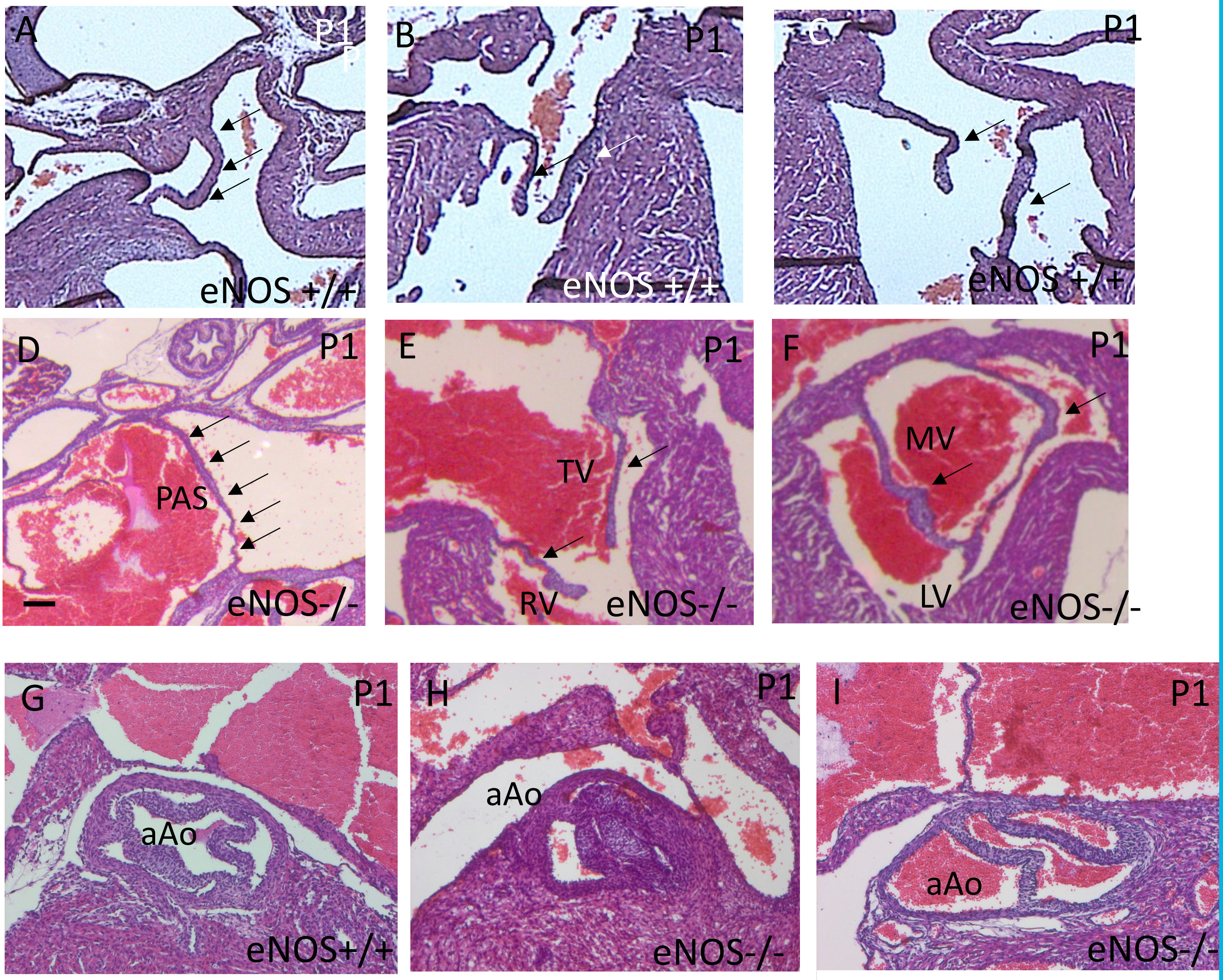

Fig. S2. Atrioventricular and arterial valves in eNOS mutants and controls. A-F) An intact primary atrial septum (arrows in A and D) and grossly normal atrioventricular valves (arrows in E,F) can be seen in an eNOS mutant, compared with a wild type
(arrows in B,C). G-I) Abnormal aortic valves (H,I), including BAV (I) are seen in eNOS mutants, compared to the normal tricuspid appearance of the aortic valve in wild type fetuses (G).
aAo = ascending aorta, LV = left ventricle, MV = mitral valve, PAS = primary atrial septum, RV = right ventricle, TV = tricuspid valve.

Scale bar = 1200μm.

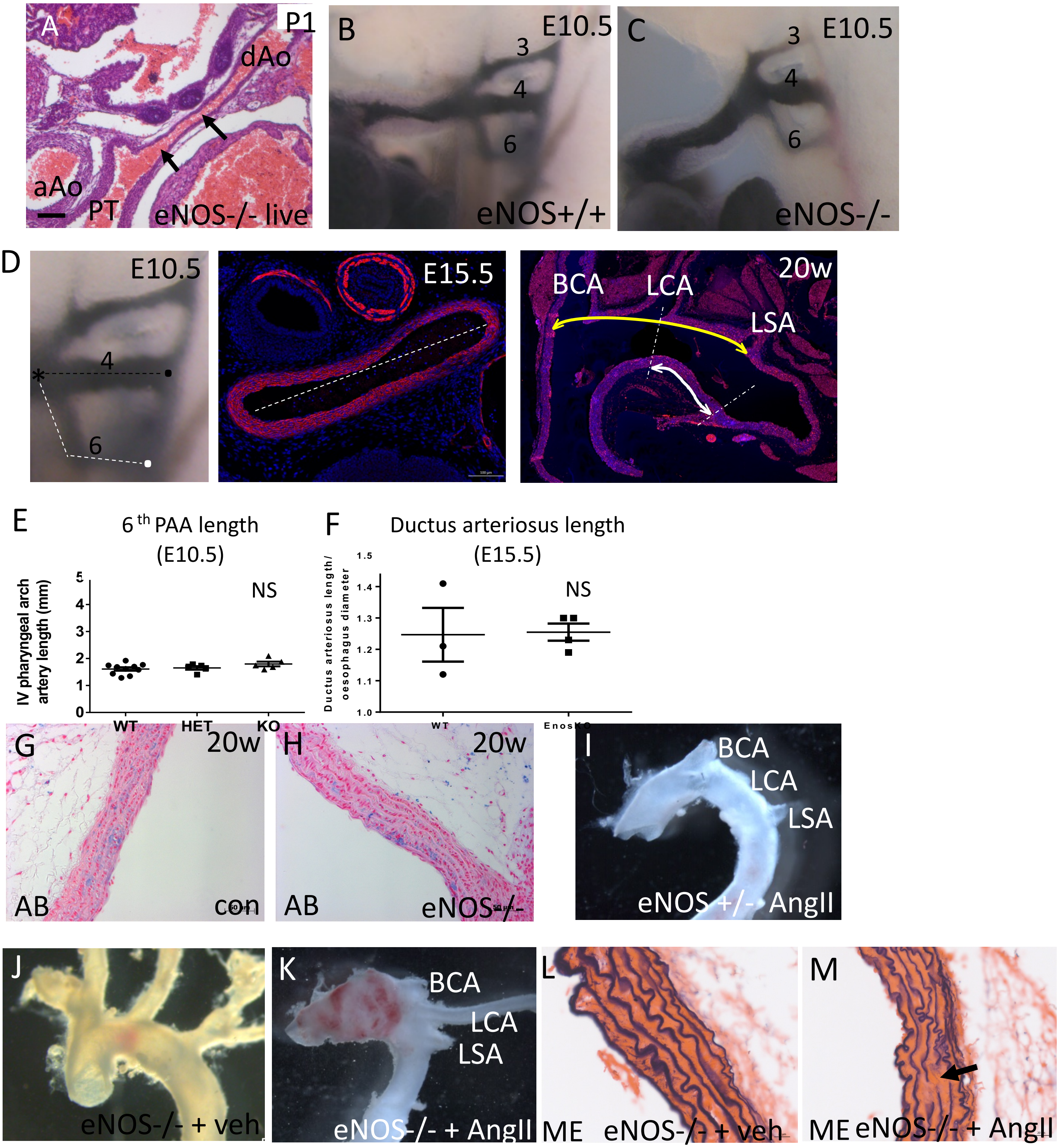

Fig. S3. Pharyngeal arch measurements and susceptibility to aortopathy in eNOS mutants and controls. A) Persistent ductus arteriosus seen at P1 in an eNOS mutant. B,C) Ink injected pharyngeal arches in eNOS +/+ and -/- at E10.5. D) Images indicating how pharyngeal and aortic arch measurements were taken at E10.5, E15.5 (dotted lines) and at 20 weeks. E,F) Neither 6th Aortic arch or ductus arteriosus length are abnormal in eNOS mutants at E10.5 and E15.5. G,H) Comparable distribution of Alcian Blue staining in control and eNOS aortic arch at 20 weeks. I-K) Administration of Angiotensin II induces aortic aneurism and dissection in the ascending component of the aorta in an eNOS mutant. L,M) whereas elastin breaks are rare in eNOS mutants treated with vehicle but are much more abundant in eNOS mutants treated with Angiotensin II (arrow in M).

aAo = ascending aorta, AB – alcian blue, AngII = angiopoietin II, BCA = brachiocephalic artery, dAo = descending aorta, LCA = left common carotid artery, LSA = left subclavian artery, ME = Miller's elastin, PT = pulmonary trunk. 3,4,6 indicates pharyngeal arch identity.

Scale bar in A = 600μm, B,C= 20μm, D = 40μm (E10.5), 80μm (E15.5), 200μm (20W), G,H = 60μm, I-K = 300μm, L,M = 30μm.

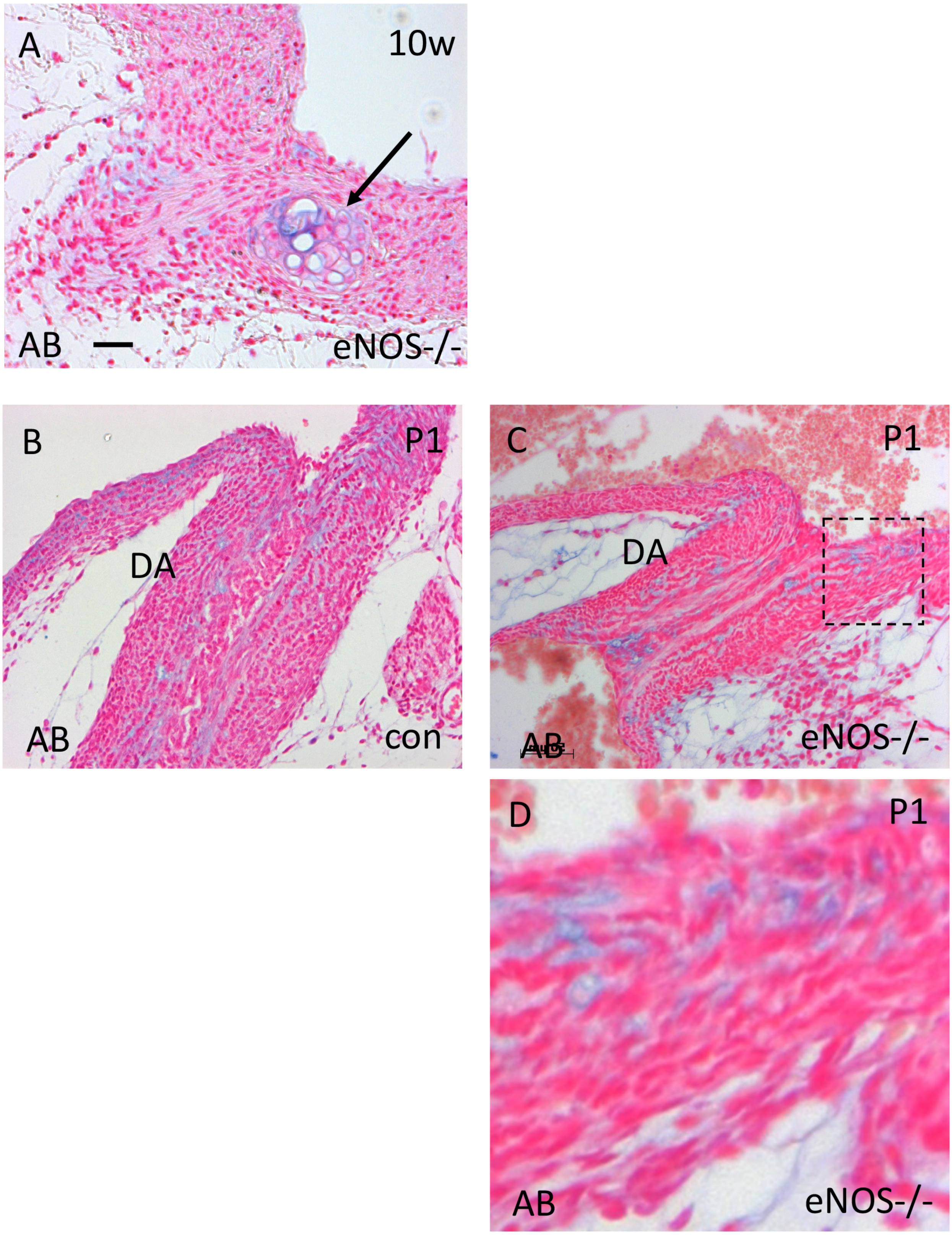

Fig. S4. Cartilaginous dysplasia develops postnatally in eNOS null mice.

A) Alcian Blue stained nodules can be seen in the periductal region of eNOS mutants at 10 weeks, but not at P1. The boxed area in C is enlarged in D.

AB = Alcian blue, DA = ductus arteriosus.

Scale bar in A = 80µm, B = 150µm, C = 100µm, D = 25µm.

Disease Models & Mechanisms • Supplementary information

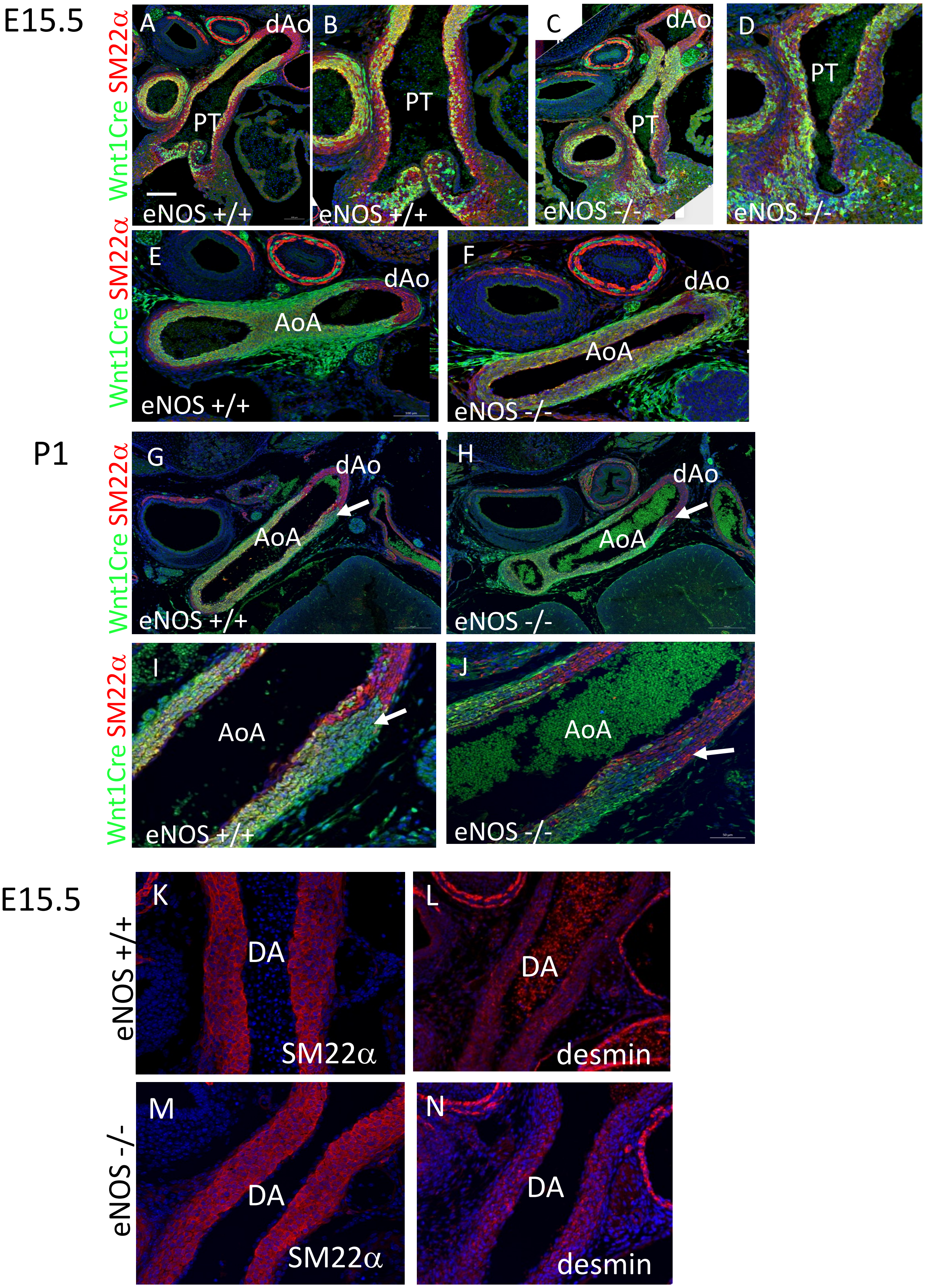
E15.5
Wnt1Cre SM22α
A
dAo
PT
eNOS +/+
B
PT
eNOS +/+
C
dAo
PT
eNOS -/-
D
PT
eNOS -/-
Wnt1Cre SM22α
E
dAo
AoA
eNOS +/+
F
dAo
AoA
eNOS -/-
P1
Wnt1Cre SM22α
G
dAo
AoA
eNOS +/+
H
dAo
AoA
eNOS -/-
Wnt1Cre SM22α
I
AoA
eNOS +/+
J
AoA
eNOS -/-
E15.5
eNOS +/+
K
DA
SM22α
L
DA
desmin
eNOS -/-
M
DA
SM22α
N
DA
desmin

Fig. S5. NCC and SMC marker distribution at E15.5 in eNOS fetuses and their control littermates. A-J) The pattern of Wnt1Cre labelling in the pulmonary trunk and ductus arteriosus and the aortic arch is similar in controls and eNOS null mutants at E15.5 and P1 although the cells appear disorganized in the pulmonary trunk. The descending aorta does not label with Wnt1Cre. An abrogation can be seen in the Wnt1Cre labelling in the eNOS mutant at P1, close to where the arch joins the descending aorta (arrow in G-J). K-N) SM22a labelling is similar in the ductus arteriosus of controls and eNOS mutants at E15.5 (K,M) although desmin expression appears increased in the mutants (L,N).

aAo = ascending aorta, AoA = aortic arch, dAo = descending aorta, DA = ductus arteriosus, PT = pulmonary trunk

Scale bar in A,C = 150μm, B,D= 50μm, E,F = 100μm, G,H = 250μm, I,J = 70μm, K-M = 50μm.

Disease Models & Mechanisms • Supplementary information

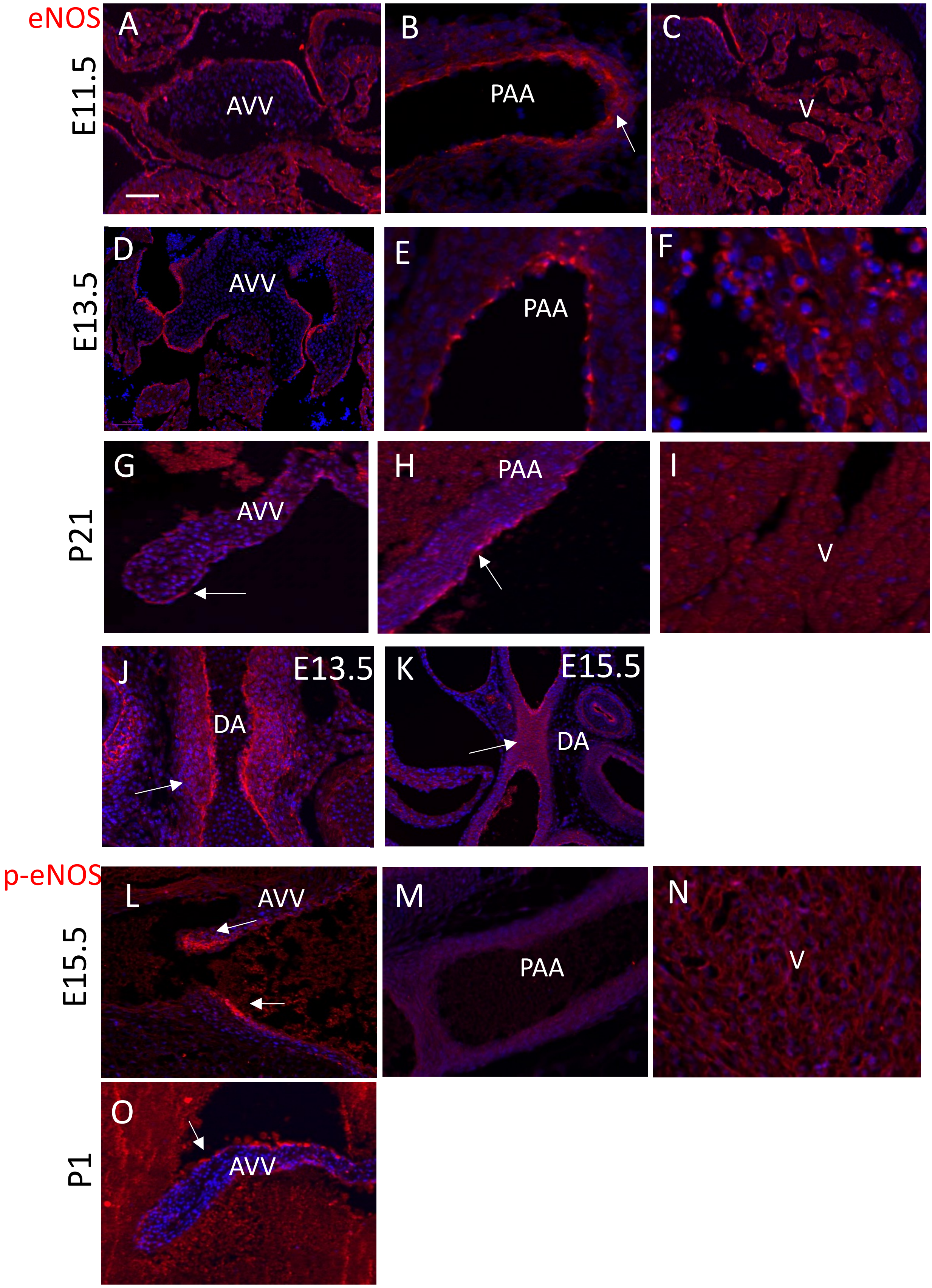

Disease Models & Mechanisms • Supplementary information

Fig. S6. eNOS and p-eNOS expression in fetal and neonatal cardiovascular tissues.
A-I) eNOS expression is expressed in the endocardium of the atrioventricular valves, pharyngeal arch arteries and ventricles at E11.5 and E15.5 but declines in the ventricles at P21. J,K) eNOS is found in the media of the ductus arteriosus at E13.5 and E15.5. L-O)p-eNOS is found in the atrioventricular valves at E15.5 and P1, but not in the pharyngeal arch arteries or ventricles.

AVV = atrioventricular valves, DA = ductus arteriosus, PAA = pharyngeal arch artery, V – ventricle

Scale bar in A,C = 150μm, B,J = 40μm, D = 120 μm, E = 75μm, F = 250μm, G-I = 400μm, K,N = 150μm, L,M,O = 100μm.

Disease Models & Mechanisms • Supplementary information

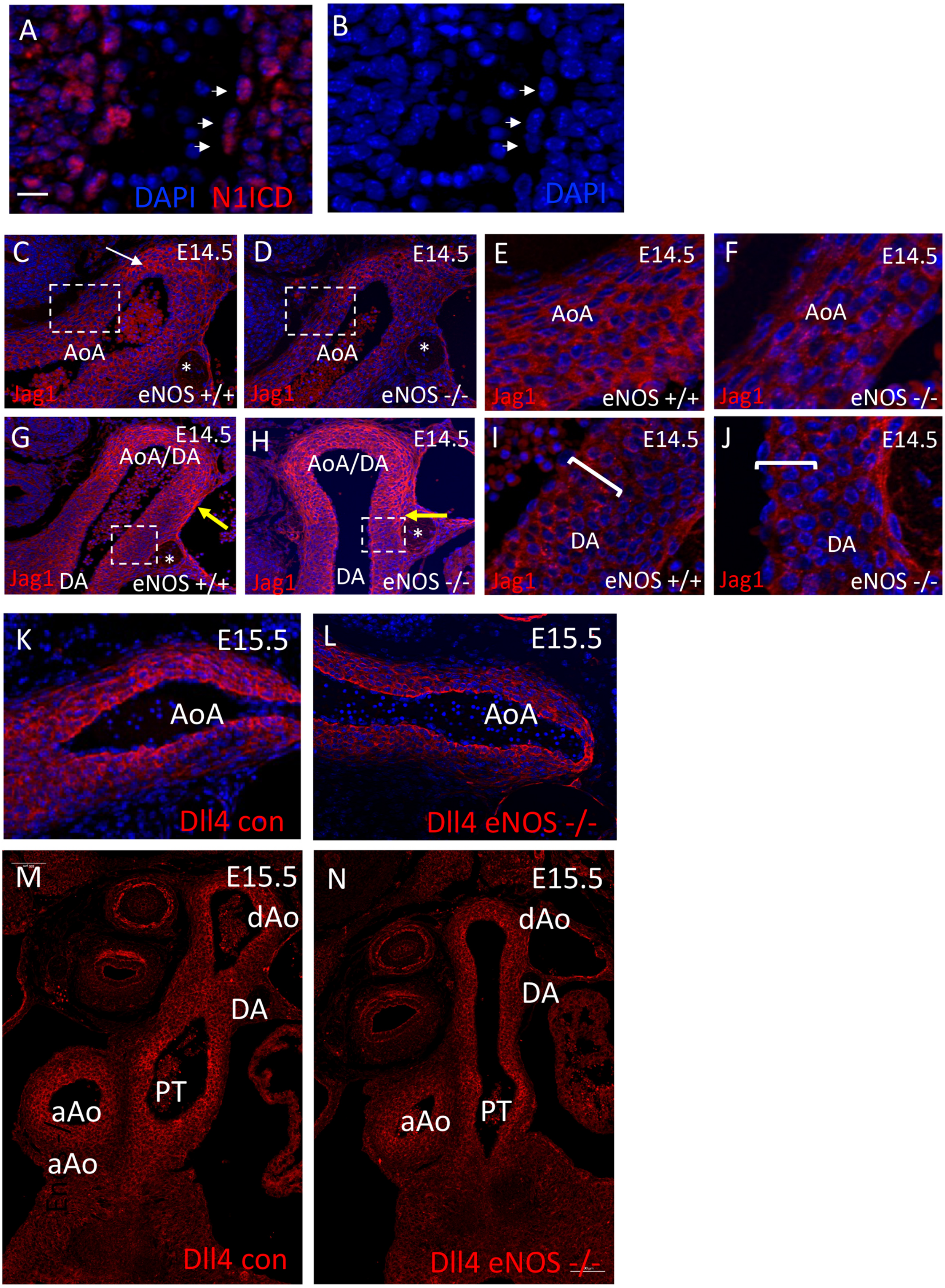
A
DAPI N1ICD
B
DAPI
C
E14.5
AoA
Jag1
eNOS +/+
D
E14.5
AoA
Jag1
eNOS -/-
E
E14.5
AoA
Jag1
eNOS +/+
F
E14.5
AoA
Jag1
eNOS -/-
G
E14.5
AoA/DA
Jag1
DA
eNOS +/+
H
E14.5
AoA/DA
Jag1
DA
eNOS -/-
I
E14.5
DA
Jag1
eNOS +/+
J
E14.5
DA
Jag1
eNOS -/-
K
E15.5
AoA
Dll4 con
L
E15.5
AoA
Dll4 eNOS -/-
M
E15.5
dAo
DA
PT
aAo
aAo
Dll4 con
N
E15.5
dAo
DA
aAo
PT
Dll4 eNOS -/-

Fig. S7. Notch1ICD, Jag1 and Dll4 expression in eNOS null embryos/fetuses and their control littermates. A,B) Notch1ICD can be seen in the flattened nuclei (arrowheads) of endothelial cells in the ductus arteriosus at E14.5. **C-J)** Jag1 is expressed throughout the media of the aortic arch including the region where the ductus arteriosus joins the arch/descending aorta, but only in the inner luminal layers of the media in the ductus arteriosus in the wild type fetus at E14.5 (white bar in I). The region where Jag1 is found throughout the vessel wall appears shifted proximally in the eNOS mutants, as seen when compared to the position of the left recurrent laryngeal nerve (asterisks and yellow arrows). Jag1 immunoreactivity appears reduced from the innermost layers of the media of the ductus arteriosus in the eNOS null fetus (white bar in J). K-N) Dll4 is expressed in the endocardium and media of both the aortic arch and ductus arteriosus but is similar in controls and eNOS null fetuses.

AoA = aortic arch, dAo = descending aorta, DA = ductus arteriosus, PT= Pulmonary Trunk.

Scale bar in A,B = 300µm, C,D,G,H = 50µm; E,F,I,J = 200µm; K,L = 100µm, M,N = 80µm.

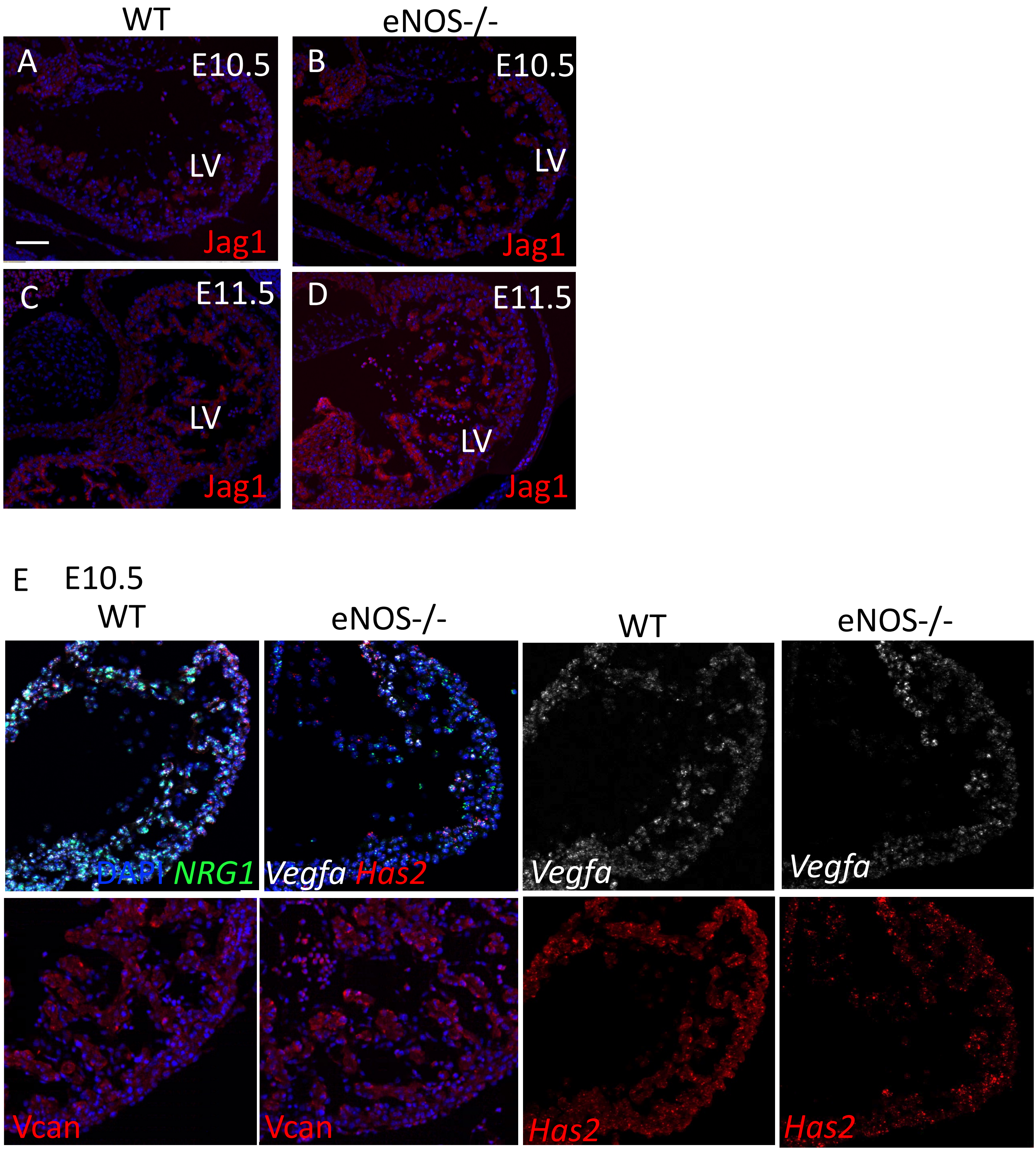

Disease Models & Mechanisms • Supplementary information

Fig. S8. Jag1, versican, *NRG1*, *Vegfa*, and *Has2* expression in embryonic ventricles from eNOS mutants and controls. A-D) Jag1 is found at only low level in the ventricles at E10.5 and E11.5 and is similar in controls and eNOS null embryos. E) At E10.5, RNAScope for *Vegfa* and *Has2* showed no obvious differences between WT and eNOS mutants. Versican immunohistochemistry was also similar between wild types and eNOS mutants.
aAo = ascending aorta, AoA = aortic arch, dAo = descending aorta, DA = ductus arteriosus, LV = left ventricle, PT = pulmonary trunk.
Scale bar = 50µm.

Disease Models & Mechanisms • Supplementary information
